# Direct evidence for processing *Isatis tinctoria* L., a non-nutritional plant, 32–34,000 years ago

**DOI:** 10.1371/journal.pone.0321262

**Published:** 2025-05-09

**Authors:** Laura Longo, Mauro Veronese, Clarissa Cagnato, Giusi Sorrentino, Ana Tetruashvili, Anna Belfer-Cohen, Nino Jakeli, Tengiz Meshveliani, Moreno Meneghetti, Alfonso Zoleo, Antonio Marcomini, Gilberto Artioli, Elena Badetti, Karen Hardy

**Affiliations:** 1 Department of Environmental Science, Informatics and Statistics (DAIS), Ca’ Foscari University of Venice, Mestre, Venice, Italy; 2 Department of Geosciences, University of Padua, Padova, Padova, Italy; 3 Department of Chemical Sciences, University of Padua, Padova, Padova, Italy; 4 UMR 7268 Anthropologie Bio-Culturelle, Droit, Éthique et Santé (ADES), Aix-Marseille Université, Marseille, France; 5 The Faculty of Law, Education, Business and Technology, European University, Tbilisi, Georgia; 6 Institute of Archaeology, The Hebrew University of Jerusalem, Jerusalem, Israel; 7 Georgian National Museum, Tbilisi, Georgia; 8 Institute of Paleoanthropology and Paleobiology, Georgian National Museum, Tbilisi, Georgia; 9 Department of Archaeology, The University of Glasgow, Glasgow, United Kingdom; University of Haifa, Zinman Institute of Archaeology, ISRAEL

## Abstract

Recovering evidence for the intentional use of plants in the Palaeolithic is challenging due to their perishable nature as, unlike chipped stone or bone artefacts, plant remains are rarely preserved. This has created a paradigm for the Palaeolithic in which plants seldom feature, resulting in a partial and skewed perspective; in fact, plants were as essential to human life then as they are today. Here, we combine morphological and spectroscopic analyses (µ-Raman, µ-FTIR) to provide robust multiscale physical and biomolecular evidence for the deliberate pounding and grinding of *Isatis tinctoria* L. leaves 34–32,000 years ago. The leaf epidermis fragments were found entrapped in the topography of the used surface of unmodified pebbles, in association with use-wear traces. Although their bitter taste renders them essentially inedible, the leaves have well-recognised medicinal properties and contain indigotin precursors, the chromophore responsible for the blue colour of woad, a plant-based dye that is insoluble in water. We used a stringent approach to contamination control and biomolecular analysis to provide evidence for a new perspective on human behaviour, and the applied technical and ecological knowledge that is likely to have prevailed in the Upper Palaeolithic. Whether this plant was used as a colourant, as medicine, or indeed for both remains unknown, but offers a new perspective on the fascinating possibilities of non-edible plant use.

## Introduction

Modern humans (*Homo sapiens*) first appear in the archaeological record around 300,000 years ago, in Africa. Most of the evidence for their cognitive and technological abilities is based on recovered assemblages of chipped stone artefacts and animal bones since these endure far longer in the archaeological record than plants [1]. Accordingly, the Palaeolithic narrative centres primarily on animal hunting and stone tool manufacture. Perishable materials, the so-called “missing majority” [2], notably plants for which there is growing evidence for their use as food [3–5], string and cordage [6], weaving [7] and medicine [8], are largely missing¸ creating a partial narrative. There is therefore, a need to identify and demonstrate the use of plants and the roles they played in a wide range of activities, many of which may still be unknown. Ultimately, this will contribute to a broader understanding of Palaeolithic behaviour.

Finding direct traces of the deliberate exploitation of plant materials is complex since macroscopic un-charred plant remains rarely survive through Palaeolithic timescales and, even when they do, their presence can be argued as natural deposits [9] or the result of bioturbation or other taphonomic events [10]. Therefore, demonstrating use of plants during human evolution requires not only conditions that are favourable to the long-term survival of plant remains, but also needs to be exhaustive, as the finds must indicate human involvement, and also offer stringent evidence to negate natural occurrence and modern contamination [11]. A lack of awareness of the potential for complex plant processing can compromise evidence for this during excavation and post-excavation processes [12]. Finally, the recent application of high-resolution imaging and biomolecular techniques to the investigation of biogenic residues from the micro to the nanoscale [13–16], is now expanding the potential for detection of a new range of evidence for the use of plants and the likely behaviour associated with this. Use of these techniques can feed into a better understanding of the complexity of human-plant relationships and expand perception of plant processing. Each time a new piece of secure information emerges it provides an extraordinary insight into our prehistoric human past and contributes to a growing awareness that our Palaeolithic ancestors had sophisticated ecological knowledge of plants.

While plant processing, including cooking and grinding, has been recognised for some time for the Upper Palaeolithic [3,17–19], the evidence for modification of plant materials has, until recently, been largely restricted to identification of macro remains, notably carbonised materials and wood [20,21], likely food items [4,5] or pitch although this is generally identified by biomolecular analysis [22–26]. Plants represent an infinite resource that provide humans not only with food and raw materials but also, due to their biomolecular complexity, with medicines, poisons, flavours, aromas, hallucinogens and dyes [8]. The ingestion of non-nutritional plants containing medicinal secondary metabolites was identified in 47,000-year-old Neanderthal dental calculus [27,28], while tentative evidence for poison 40,000 years ago was recovered from Border Cave, South Africa [29]. Medicinal plants are reported from a number of Palaeolithic sites in the Caucasus [30]; however, it is challenging to demonstrate that these were ingested and/or intentionally processed [8]. To date, there is no evidence for the extraction of dyes from organic materials in the Palaeolithic; the known colourants (red, yellow, black and white) are all pigments of mineral origin apart from charcoal [31]. They are highly resistant to ageing, with little apparent degradation and known to have been used in Palaeolithic art and for other purposes. For example, ochre is known in various applications such as tanning leather or skin [32], as a preservative [33,34], as insect deterrent and as skin protection [35].

One source of evidence for the use and intentional processing of plants comes from Ground Stone Tools (GST) [3,17–19]. The use-wear traces on their utilized surfaces and the extraction of micro-residues trapped within their surface are compared to modern reference materials to reconstruct the type of materials being worked. These residues can be extracted, imaged and chemo-profiled [12,16,19].

During the functional study of GSTs from the Upper Palaeolithic site of Dzudzuana cave (Georgia, Caucasus), plant micro-residues in association with use-wear traces were identified microscopically. Some of these residues consisted of blue coloured fragments that were studied by combining physical and biomolecular methods. At the molecular level, we identified indigotin, the precursors of which are present in the leaves of indigo-bearing plants.

Here, we share the robust data derived from the chemical-physical analyses that led to the identification of the indigotin molecule, which has not previously been recognised in a Palaeolithic context. We also investigate the processes that may have led to the presence of the blue residues on the stone pebbles. To address this, we examined possible accidental or deliberate reasons for their occurrence as part of the mechanical processing of leaves, including whether the effect of releasing the precursors of indigotin - a well-documented practice in both ethnographic and historical records [36,37] - could have been deliberate. This paper is therefore focused on the recovery and identification of indigotin as an identifying feature of *Isatis tinctoria* L., extracted from the working surface of these archaeological GSTs. Finally, we provide an exhaustive explanation of the method used for extraction of the residues to ensure the samples were free of contamination during this process. While we present some preliminary use-wear observations to support the actual use of these pebbles, an exhaustive functional analysis will be the object of a separate study.

Indigotin derives from the processing of indigoid plants, in particular species within the *Isatis, Indigofera,* and *Persicaria* genera. When the leaves are crushed the precursors are released and the fermentation process begins [38–41]. Among these, *I. tinctoria*, a non-nutritional plant, is the only species native to Central Asia [42,43], whereas all the other species are of tropical origin and do not naturally grow in cool environments [36]. Metabarcoding techniques applied to the sediments from Aghitu 3 cave in Armenia (39–24,000 years old) identified *Isatis* sp., hence providing genetic evidence that the genus was present during this period in the Caucasus [14].

*I. tinctoria*, colloquially known as dyer’s woad, is a biennial plant of the Brassicaceae family that grows in dry and sunny locations. This plant is a source of indoxyl glycosides - precursors of the blue chromophore indigotin and its red isomer indirubin - naturally contained in cell vacuoles of the leaf epidermis, together with a minor quantity of flavonoids [40,41,44]. It is also well known for its medicinal properties due to the significant presence of further indole alkaloids in its roots and leaves [39,45,46].

Today, indigotin is widely used to colour cloth, in particular in the dyeing of blue jeans, which are usually manufactured from cotton (*Gossypium* sp.). To test for recent contamination, we created a reference collection of modern contaminants including cotton. Following exhaustive comparative analysis of these materials, we can confirm definitively that the residues retrieved on the GSTs from Dzudzuana were not from modern contaminants, including cotton.

We developed an innovative and robust research design that integrates synchrotron-based micro-CT scanning (to demonstrate the porosity of rocks enabling retention of residues), microscopy at various resolutions (to document the presence of residues and their correlation with modifications to the stone surface caused by mechanical processing), and spectroscopy techniques, in particular Raman and FTIR (Fourier transform infrared) (to characterise residues and the chemical profile of their constituent molecules). We demonstrate that *I. tinctoria* was pounded at Dzudzuana by *Homo sapiens,* 34,000 years ago, using unmodified river pebbles. Unmodified pebbles are recognised as tools in the Upper Palaeolithic, although to date they have been associated with the processing of edible plants [3,16,18,19,47] and inorganic materials such as ochre [48,49]. We do not claim to understand why this plant was being ground up, however we acknowledge that it is well known today for its medicinal and blue dye applications [36,38]. By demonstrating that plant processing was not restricted to edible plants, our research provides a new dimension to human behaviour and contributes significantly to the growing body of evidence highlighting the complexity of human-plant interactions in the Upper Palaeolithic.

## Materials

**Fig 1:**
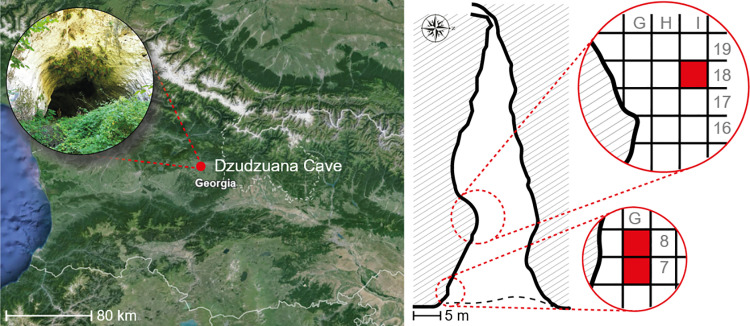
Area under study. Left: Location of Dzudzuana cave (modified after Google Earth) and cave entrance (Image by LL). Right: map of the cave with the excavated area inside the red circles (redrawn and modified from Bar-Yosef et al. 2011, p. 334, Fig 2 [50]), red squares show where the six pebbles were retrieved from. Excavations in squares G-7, G-8 and I-8 reached the full depth of the archaeological sequence. These are also the squares where the stone pebbles were retrieved.

**Fig 2:**
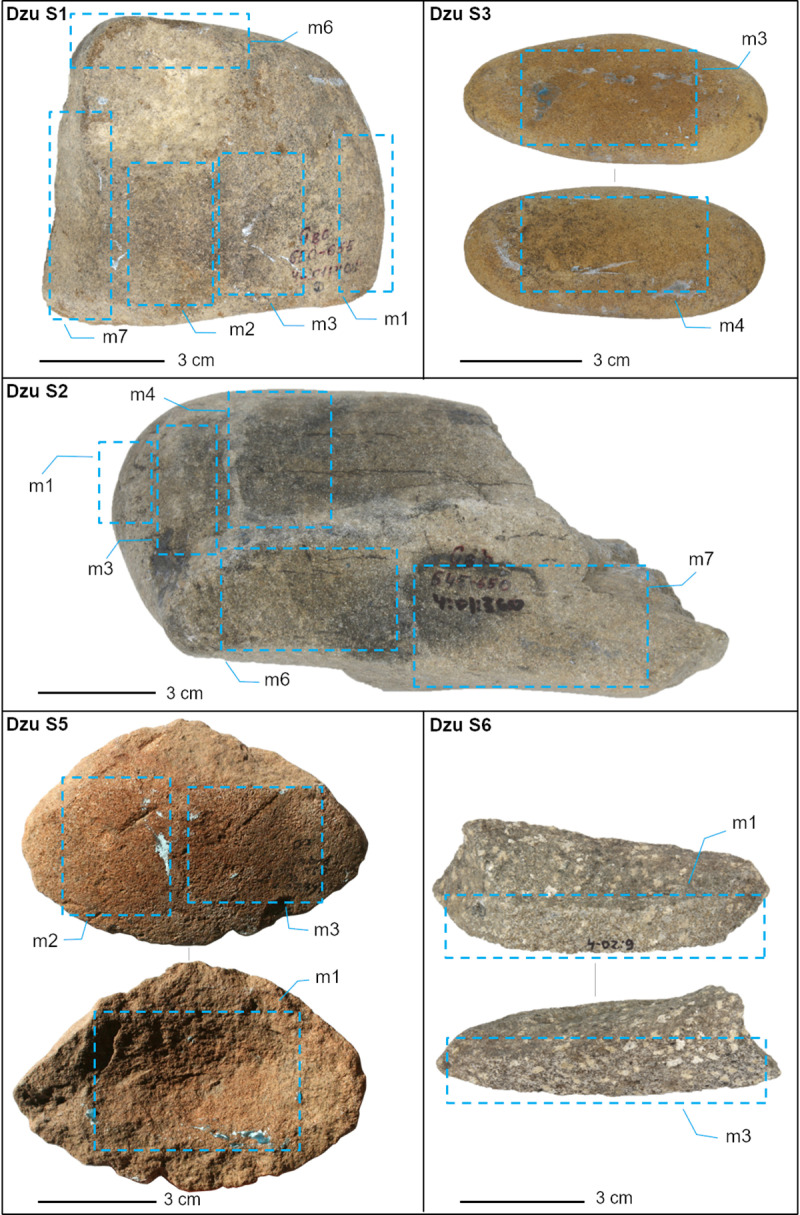
Out of the six stone pebbles retrieved in Unit D, here are imaged five archaeological pebbles showing the position of the analysed moulds indicated by blue dashed lines, and from which micro-residues have been extracted and use-wear traces have been identified.

Dzudzuana cave, Imereti (Western Georgia), is an Upper Palaeolithic site (Fig 1) comprising three discrete Palaeolithic units spanning ca. 20,000 years of occupation, the lowest unit (D) dating to 34.5–32.2 cal. BP [50] (Fig 1). The archaeological material from this unit comprised chipped stone assemblages, faunal remains, bone tools including a needle, as well as engraved and pierced animal teeth likely to have been used as ornamental artefacts [50]. In addition, the findings included six river pebbles which are the focus of the present study (Fig 2; S1 Fig and S1 Table). Five of these pebbles provide clear evidence of use in the form of functionally active areas and associated micro-residues.

During this period, several climatic fluctuations occurred; however, during the human occupation, the climate at Dzudzuana cave was generally mild, with species such as wild grape (*Vitis sylvestris*), hazel (*Corylus* sp.), oak (*Quercus* sp.), beech (*Fagus sylvatica*), birch (*Betula* sp.) and *Artemisia* sp. all present in the Caucasus [50]. Moreover, several Brassicaceae species, among which *Isatis* sp., was detected by metabarcoding [14]. The present study of residues adhering to GSTs supported by preliminary use-wear analysis, demonstrates the purposeful use of these stone pebbles, the archaeological integrity of the material recovered and its accurate identification (Methods, S1 and S3 Figs, Table 2).

**Table 2:** The sequential sampling procedure.

In the field			
1	Stone tool provenience	Different field seasons 2002–2007. Different squares: G 7, G 8, I 8 (Fig 1). Unit D.	
2	Rinsed	Running water in the Nikrisi River.	
3	Labelled	Ink (no varnish).	
4	Bagged	Individually in paper bags.	
5	Transported	To the Georgian National Museum (GNM), Tbilisi (Georgia).	
**Museum Storage conditions** ^**a**^			
1	Handling	No previous handling or processing of the stone pebbles is recorded since the year of retrieval they entered the GNM collection, until 2022 when they were accessed for the present study.	
2	Placement	Since entering the GNM storage facilities each bagged stone was placed in a drawer inside a wooden cabinet.	
3	Temperature	Room temperature.	
**Sampling equipment prepared at DAIS - Ca’ Foscari University and shipped to the GNM**			
1	Cleaning		All utensils (e.g. glassware, petri dishes, metal tools) were washed with the surfactant soap (Contrad®), bleached and UV irradiated.
2	Packaging		Each tool was sealed in a zip-lock bag.
**Sampling activities at the GNM (2022)**			
1	Standard preparation		Prior to sampling, the desk surfaces were routinely bleached and covered with a starch-free plastic cloth that was changed daily.
2	Handling		Stone tools were handled with nitrile starch-free gloves, renovated frequently during the sampling.
3	Cross-control: Petri dishes		To collect potential contaminants, traps consisting of Petri dish filled with ultrapure water were set on desks, shelves, and in the vicinity of the sampling area. The Petri dishes were set during all the stages of the described procedure: i.e., in the Museum, in the laboratory and during microscopy observation.
4	Cross-control: Paper bags		Dust covering the paper bags was collected.
5	Cross-control: Stone tools		The surface of the stone pebbles, with lumps of soil still adhering, was softly brushed to remove modern dust.
6	Documentation/imaging		Stones were documented through imaging and photogrammetry.
**Laboratory of Palynology of the Palaeoanthropology and Paleobiology Research Institute of the GNM**			
1	Cleaning		Prior to sampling, the desk surfaces were routinely bleached. Traps were placed in the immediacy of the working areas.
2	Stone tool residue sampling		For sonication each stone was placed in a zip-lock bag, filled with ultrapure water. The dispersion resulting from sonication was centrifuged. Once sonicated the pebbles were set to dry under a fume hood, loosely covered with aluminium foil.
3	Moulding		The surface of the pebbles was moulded with PVS. Each mould was immediately bagged. Moulds were taken of ventral, dorsal, and also lateral sides of the stones as cross reference (Fig 2). Two moulds were taken at each location: the first peel removed any remaining sediment on the pebble surface while the 2^nd^ recovered residues entrapped deep inside the stone pockets (Supplementary Figs 2 and 3).
**Processing at DAIS under lab-controlled conditions**			
1	Cleaning		Prior to processing, the desk surfaces were routinely cleaned with surfactant soap (Contrad®). Traps were placed in the immediacy of the working areas.
2	Extracted samples (from GNM)		Processing as reported in Cagnato and Ponce, 2017; Longo et al. 2021, 2022; Birarda et al. 2023.
3	Mould subsampling		Subsampling of the 2^nd^ moulds carried out under fume hood (S4 Fig).
4	Sonication		Sonication of the moulds (S4 Fig).
5	Slide cleaning		Borosilicate slides and coverslips were washed with Contrad® and rinsed with ultrapure water. Slides were kept in a 50mL tube filled with ethanol.
6	Deposition on the slide for OM/SEM observation		0.05 µL droplets of the obtained solution was placed on the borosilicate slide previously cleaned and stored as described above.
**Control samples**			
1	From the site		Lump of sediment still adhering to the stone pebbles; Sediment from Unit D. Stones collected from the cave floor surface. Stones collected in the Nikrisi River (also used in replicative experiments).
2	During sample processing		Petri dish content. Dust covering paper bags. Modern fabrics (blue jeans).
3	Analysis		All samples shipped to DAIS - Ca’ Foscari University.

^a^The conditions applied are reported in Longo et al. 2022.

### Stone pebbles biography and taphonomic history

Here we report on the methodological sequence during the different stages of the Dzudzuana stone pebbles analysis. The sequential sampling procedure applied is summarized in Table 2 (Methods) and follows the procedures previously established and published by the authors [11,12,16,18,51].

### Processing during field-work

Six stone tools were identified during several archaeological excavation campaigns (2002–2007, details are reported in S1 Table). The pebbles were retrieved in different squares (G-7, G-8, I-8; Fig 1) from Unit D, excavated by 5 or 10 cm levels. Three pebbles (Dzu S1 to Dzu S3) derive from the deepest portion of Unit D, excavated in 2002, whereas three samples (Dzu S4 to Dzu S6) come from the uppermost part of the same unit, excavated in 2007. Only two pebbles are complete (Dzu S3 and Dzu S4). Dzu S1, Dzu S2, Dzu S5, and Dzu S6 are fragments of larger pebbles; Dzu S5 still retains part of the cortical surface and Dzu S6 is a mid-segment of a pebble. During field-work after their retrieval, each pebble was summarily rinsed in running water. Each stone was inventoried in the log book of the excavation and labelled according to museum accession number, and finally placed individually in a labelled paper bag. The stones were then taken to the Georgian National Museum (GNM, Tbilisi).

### Museum storage

The stone pebbles were catalogued, then stored in the museum storage unit, placed in a drawer inside a wooden cabinet and kept at room temperature. The drawers were not opened until the present study.

### Preparation of materials employed during the sampling

All the labware utensils used during the sampling were washed, bleached, UV irradiated, and bagged individually at the Department of Environmental Science, Informatics and Statistics (DAIS, Ca’ Foscari University of Venice, Italy). The materials were then sealed and shipped to GNM.

### Sampling in the Georgian National Museum

The six stone pebbles were accessed in 2022 at the GNM. One of them shows a morphology highly unlikely to be involved in pounding or grinding activity (namely, Dzu S4). Hence, five stone pebbles were considered for further analysis. They were always handled with powder-free gloves, and the gloves were frequently replaced during handling. Processing of the artefacts was carried out in the storage unit. The desk surfaces, on which the pebbles were placed were routinely bleached and covered with a starch-free plastic cloth that was changed every day. Petri dish traps filled with ultrapure water were set on desks, shelves and in the vicinity of the sampling area to collect potential contamination.

The paper bags containing the stones pebbles were dusted on the outside, and the sweepings were retained (see Methods, Table 2). Then, each pebble was carefully pulled out, very softly dusted with a clean brush, and the collected dust was also retained for cross-control.

Stones were documented through imaging and photogrammetry using a digital microscope brought into the storage unit. Finally, each pebble was re-bagged in clean plastic bags.

### Laboratory of Palynology of the Palaeoanthropology and Paleobiology research institute of the GNM

After this first stage of the processing, all the following preparation steps were carried out under controlled lab-conditions in the Laboratory of Palynology of the Palaeoanthropology and Paleobiology Research Institute, GNM, Tbilisi. The naked-eye observations of the pebbles revealed visible small lumps of soil still adhering to their surfaces. To remove this, the stones were individually placed in zipped bags containing ultrapure water and then submerged in a sonication bath (Elma S6OH, Elmasonic) for 1 hour and 30 minutes. The obtained solution was poured into tubes (50 ml) and centrifuged for 10 minutes at 3000 rpm (LMC 3000, laboratory centrifuge, Biosan). The solution was then transferred into 2 ml vials and a few droplets of ethanol (EtOH, Merck, gradient grade) were added to avoid biogenic activity (fungi and bacteria). After sonication, the stones were left to dry, loosely covered with aluminium foil, under a fume hood.

Once dry, selected areas of the stone surfaces, comprising visually evident functionally active areas, were moulded (see Fig 2 for the placement of the moulds) following a well-established procedure [12,18,52–54]. Moulding is a long-accepted method used widely by functional analysis specialists to obtain detailed negative copies of the surface, especially useful in contexts where necessary facilities or equipment to conduct full analyses are unavailable [47,53–56]. The moulding material is a silicone-based dental impression polymer, polyvinyl siloxane (PVS) (Coltene®; Speedex, Universal Activator, with linear shrinkage = -0.2%) [56].

**Fig 3:**
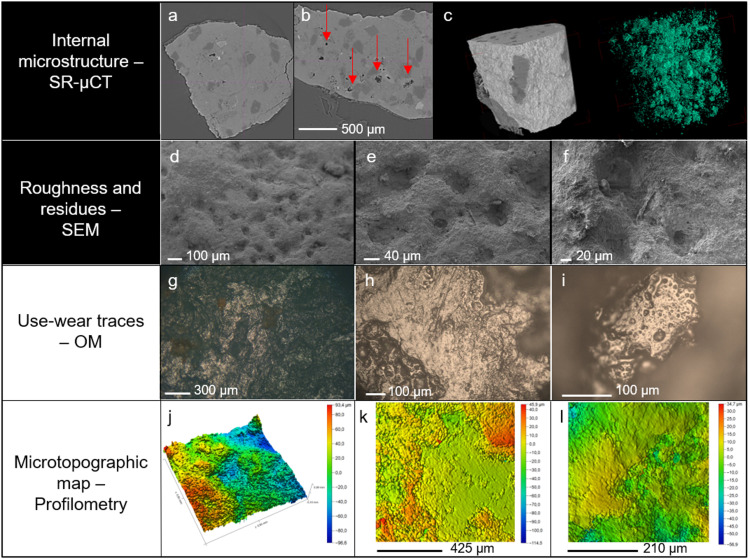
Microstructure and surface alterations of the pebble stones. Two upper rows: modern reference pebbles collected from the Nikrisi River. Panels a-c: SR-μCT of a fragment sampled from a reference pebble; a and b: slices of the inner structure showing the pits and cracks (indicated by the red arrows); c: 3D reconstruction of the sample volume with overall microporosity shown in green. Panels d-f: surface roughness of the reference pebbles used in the replicative experiments as observed with the SEM (WD 2-3 mm, 4-5 kV), highlighting the presence of micro residues including a fibre entrapped in pits or cracks (f). Two lower rows: moulds of the archaeological pebbles Dzu S1, Dzu S2 and Dzu S5. Panels g-i: examples of use-wear traces including flattened areas, polish and striations as observed under the metallographic microscope. Panels j-l: microtopography of sampled areas as imaged by confocal profilometry. The false-colour maps show the pebbles’ surface texture, highlighting the presence of pits (j) as well as flattened areas and striations (k and l).

Moulds of dorsal and lateral sides of the stones, seemingly without evidence of use, were also taken for cross reference. Two successive moulds were taken at each location, with the first removing any remaining surface sediment while the second recovered residues entrapped deep within the stone cracks as per the established method [11,12,18,51,54] (Fig 3, S4 Fig). Each mould was immediately bagged individually. Due to the PVS peel-off effect, the moulds can also dislodge residues entrapped within the pockets (pits or cracks) of the coarse stone surfaces [12,16,18] allowing the recovery of different micro-residues entrapped within these (S2 and S4 Figs).

### Processing at DAIS under lab-controlled conditions

At DAIS, moulds were initially examined under a fume hood. The mould observation revealed the presence of some residues still adhering to the stones in both the first and second moulds, suggesting that they remained entrapped deep inside the pockets on the surface of the pebbles (S2 Fig). The first mould removed the residues nearest the surface, while the second mould extracted the deeper ones. Therefore, the residues extracted with the second mould had been protected from exposure to outside elements, and it is for this reason that we examined only their internal (2^nd^) moulds.

The 2^nd^ moulds from the five relevant pebbles (Dzu S1, Dzu S2, Dzu S3, Dzu S5 and Dzu S6) were subsampled and cut in half. One half was immediately stored for analysis of wear-traces, while each other half was individually placed in a test-tube with 40 ml of ultrapure water. The tubes, set in an ice bath, were sonicated using an inserted ultrasound probe (UP-200S Hielscher Ultrasonics GmbH, Germany) operating at 100 W for 10 min (0.5 cycle, 80% amplitude) for cycles of 3 minutes (for a total of 12 minutes). The ultrasound probe was carefully cleaned before and after each sonication. The obtained samples were then processed according to the established procedures [12,57], and the extracted micro residues underwent imaging and chemo-profiling (Methods, Table 1).

**Table 1:** Summary of the analytical techniques utilised and samples investigated.

Equipment	Facility	Operational Modality	Type of samples studied
Nikon D3400 Objective Nikkor 18-55 mm, 1:3–5, 6 G	Georgian National Museum, Tbilisi (Georgia)	Iso A-320, f4 Image dimension: 6000x4000 px	Stone pebbles
Leica DCM 3D profilometer	MUSAM-Lab, IMT School for Advanced Studies, Lucca (Italy)	Confocal mode Lens: 10× and 20×	Moulds
Wild M3Z	IUAV-LAMA, Venice (Italy)	Stereomicroscope (Reflected and Transmitted light) Magnifications: 10–40×	Moulds (archaeological and replicative pebbles); Experimental pebbles; Modern plant fibres
Leitz DMRXP	IUAV-LAMA, Venice (Italy)	OM (Reflected, Transmitted and Polarised light) Magnifications: 50×, 100×, 200×	Moulds (archaeological and replicative pebbles); Residues (archaeological and from replicative experiments)
Leica DMIL LED	DAIS, Ca’ Foscari University of Venice (Italy)	OM (Transmitted and Polarised light) Magnifications: 200×, 500×, 1000×	Residues (archaeological and from replicative experiments)
3D Measuring Laser Microscope LEXT OLS 4000	Geosciences Dept., University of Padua (Italy)	Confocal Scanning Microscopy (CSM) Magnifications: 200×, 500×, 1000×	Moulds Residues (archaeological and from replicative experiments)
Zeiss EVO 15	IUAV-LAMA, Venice (Italy)	SEM Thermionic Lanthanum Coating with Pd Magnifications: 400×-2000×	Moulds Residues (archaeological and from replicative experiments)
SR- μCT	SYRMEP beamline, Elettra Synchrotron, Trieste (Italy)	Propagation-based phase-contrast SR-μCT	Replicative pebbles
Renishaw inVia Raman microscope	Chemical Sciences Dept., University of Padua (Italy)	µ-Raman spectroscopy Backscattering acquisition mode 300 mW power diode laser, exciting at 785 nm 1200 g/mm diffraction grating 50× focusing objective Peltier cooled CCD Si detector	Residues (archaeological and from replicative experiments)
Lumos II FTIR Microscope	Chemical Sciences Dept. University of Padua (Italy)	µ-FTIR Spectroscopy Reflectance acquisition mode liquid nitrogen cooled Mercury Cadium Telluride (LN-MCT) detector 4 cm^-1^ spectral resolution	Residues (archaeological and from replicative experiments)

### Control samples

Different control samples were collected during the sampling process at GNM and a stringent procedure was followed to avoid possible contamination (Methods, Table 2). During all stages of the processing, Petri dish traps were placed on the desk, on the shelves, and in the fume hood, until the stone pebbles sampling was finalized. Then, all the control samples were sealed in vials and shipped to DAIS, where they underwent optical microscopy for blue micro-residues. Control samples of dust swept from the surface of the paper bags and stone pebble surfaces were also collected.

Moreover, in order to document environmental conditions not necessarily tied to the sampling, sediment was also collected from a clean section of Unit D, rocks from the cave floor, and pebbles from the Nikrisi River. All were shipped to DAIS in Italy for further analysis where we tested for the presence of blue residues containing indigotin and compared them with the micro-residues extracted from the 2^nd^ moulds. The screening of these samples does not reveal the presence of any blue residues containing indigotin.

The pebbles collected from the Nikrisi River, selected to be morphologically similar to the archaeological ones, were also used in the replicative experiments. Finally, several modern fibres, including blue jeans fabric were sampled and used for comparison during this study.

## Results

The analysis of thin sections from the archaeological stone pebbles indicated that these are mafic igneous rock. However, since the available fragments from some of the archaeological pebbles were too small to obtain significant data on the overall stone structure, the full petrographic analysis (being invasive) was performed on pebbles collected from the adjacent Nikrisi River that crossed the limestone massifs of the Zemo Imereti Plateau [58]. The analyses of the thin sections from three river pebbles confirmed that these were epidosite and micritic limestones, consistent with the petrographic analysis of the archaeological pebbles.

X-ray Computed μ-Tomography aided with synchrotron brilliance (SR-μCT) was used to analyse the porosity of the stone pebbles collected in the river. The SR-μCT showed significant internal porosity on these igneous rocks (Fig 3 panels a-c). Moreover, microscopic study of their surface texture, investigated by means of optical microscopy (OM) and scanning electron microscopy (SEM), demonstrate that the stone is rich in irregularities and in particular microcavities where residues can become trapped (Fig 3 panels e-f).

Based on an extensive experimental programme, we can confirm that micrometric residues can become entrapped in this type of pores during the pounding of the leaves [59,60] (S1 Video).

Selected moulds from Dzu S1, Dzu S2, Dzu S3, Dzu S5, and Dzu S6 (Fig 2) were analysed according to established use-wear procedures [11] (S2 Fig), by means of OMFig 3g-3i), SEM (S3 Fig panels e,f), Laser Scanning Confocal Microscopy (LSCM, S3 Fig panels a-d) and confocal profilometry (Fig 3 panels l-n) [59]. The most common features observed are polished and flattened areas, scratches and striations (Fig 3g-3l, S3 Fig).

### The micro-residues morphology

**Fig 4:**
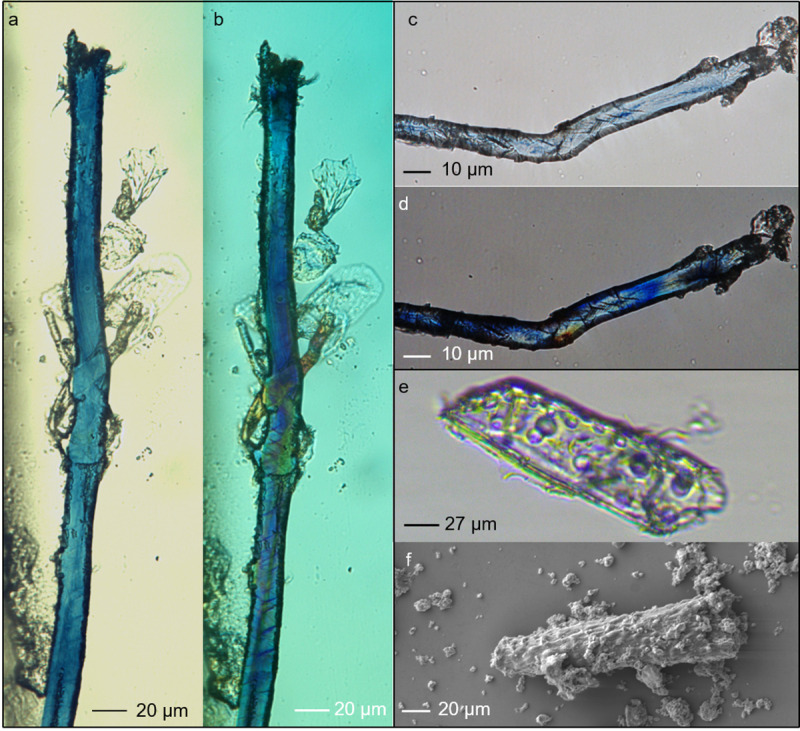
Archaeological elongated blue micro-fragments extracted from the innermost 2nd mould. Micrographs observed with OM in transmitted brightfield and cross-polarised light show nodes and kink-bands/dislocations (a: Dzu S1, mould m2; b-c: Dzu S1, mould m7; d-e: Dzu S5, mould m1), e-f: archaeological trichome showing the characteristics bumps (OM and SEM).

**Fig 5:**
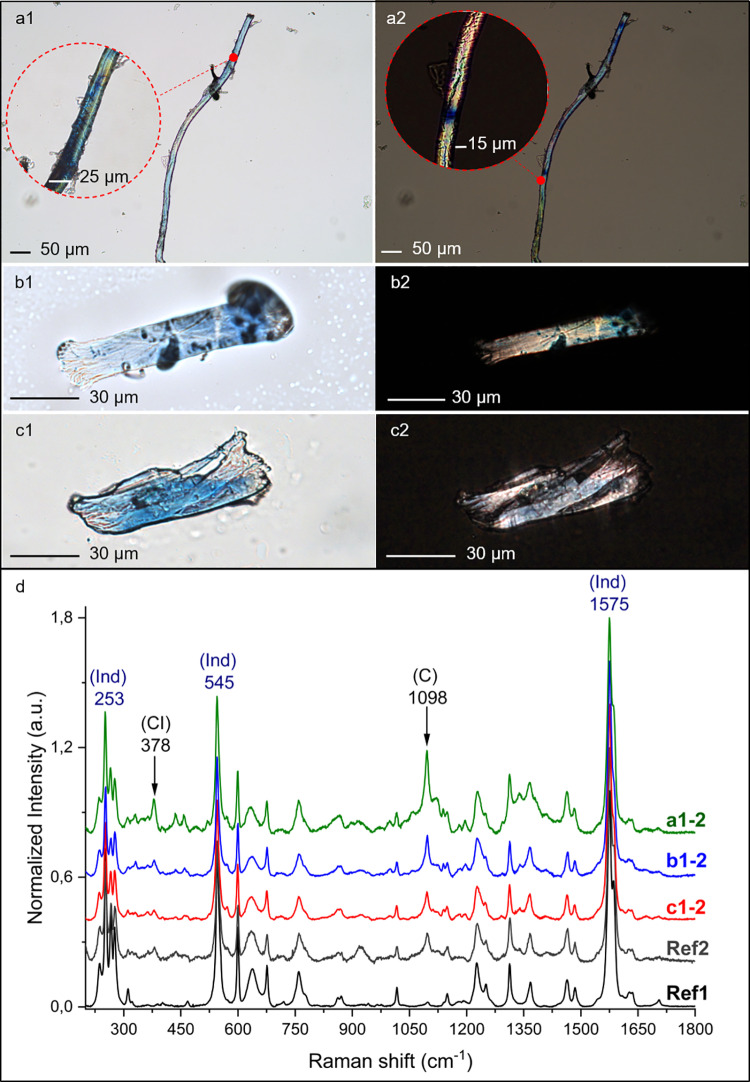
Blue residues retrieved from the Dzudzuana pebbles. Panels a-c: micrographs observed with OM in transmitted brightfield (a1, b1, c1) and cross-polarised light (a2, b2, c2) of the residues extracted from Dzu S1, Dzu S6 and Dzu S5 respectively. Panel d: µ-Raman spectra excited with a laser line at 785 nm, of the above-mentioned archaeological residues, compared with *I. tinctoria* blue residues obtained from the mechanical processing of modern leaves (Ref 2), and with the reference commercial indigotin (Ref1). The spectra have been normalised and the luminescence background has been removed. Characteristic bands of indigotin (Ind) and cellulose (C, CI) are indicated by their Raman shift.

**Fig 6:**
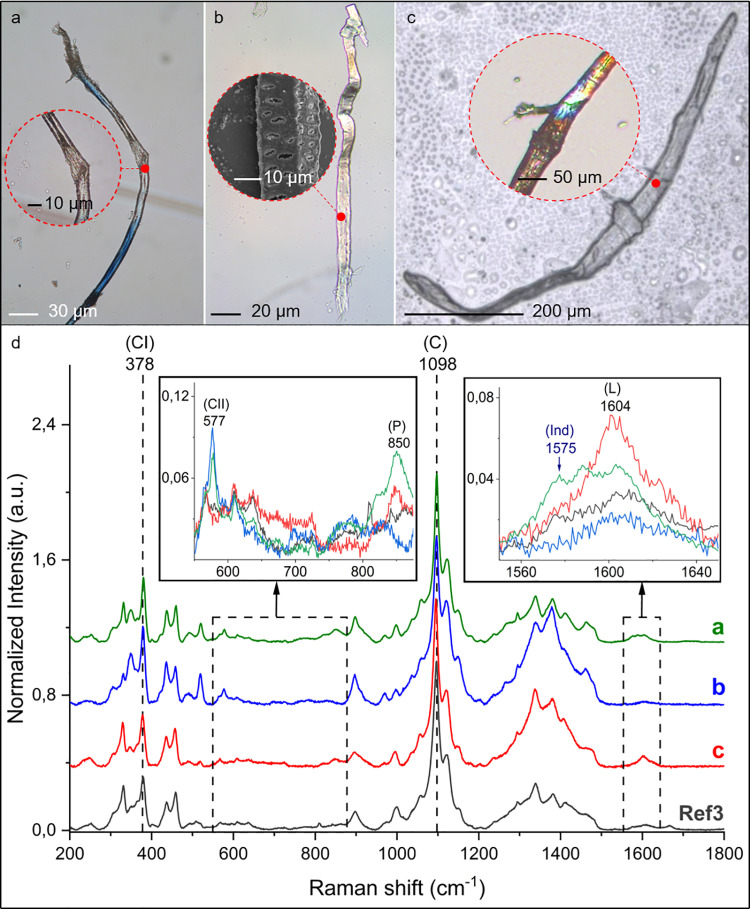
Micro-residues retrieved from the Dzudzuana pebbles. OM, LSCM and SEM images (a, b, c), and µ-Raman spectra (d). Panel a: blue micro-fragments, retrieved from Dzu S5, exhibiting non-coloured regions (OM). Panels b-c: vascular elements retrieved from Dzu S6; b: vascular tissue with aligned pits (OM) and a zoomed region as observed by SEM, retrieved from Dzu S5; c: fusiform (spindle-shape) structure tapering at each end (LSCM) and the nodes displayed in the insert (OM) retrieved from Dzu S5. Panel d: µ-Raman spectra of the micro-residues shown in the micrographs (a: spectrum of a non-coloured region of the blue fragments; b and c: spectra of non-coloured micro-residues), compared with non-coloured *I. tinctoria* fragments obtained from the processing of modern leaves (Ref3). The spectra have been normalised and the luminescence background has been removed. Characteristic bands of constituting molecules such as cellulose (C), and characteristic signals of its polymorphs (CI, CII), pectin (P), lignin (L) and indigotin (Ind) are indicated by their µ-Raman shift. Details of the spectra are reported in S14 Fig.

The solution obtained through sonication of the 2^nd^ moulds from five of the six the analysed stone tools was processed to extract micro-residues according to Cagnato and Ponce [57] (S2 Table, S4 Fig). Being aware of potential contamination issues and false positives, we deliberately decided to analyse the micro-residues from the 2^nd^ moulds, considered less prone to contamination. The morphological analysis of the micro-residues was carried out using OM, LSCM and SEM. We recovered 67 individual micro-residues (both coloured and non-coloured) that we were able to identify as of plant-based origin. Of these, 25 individual micro-residues were blue (Figs 4, 5 panels a1-a2, b1-b2, c1-2 and 6a, S5 Fig), while 42 were non-coloured (Fig 6 panels b, c, S6 Fig). Here, we focus on the results of the physical-chemical characterization of the 25 micro-residues that showed a blue colour.

On the basis of their anatomical structure, the observed residues are identified as small fragments, primarily of leaf epidermis. Esau [61] demonstrates that the structure of the stem, leaf, and root is quite similar, whereas the arrangement of vascular and ground tissues (parenchyma and collenchyma) is distinctive being permeated by a network of interconnected veins that diffuse extensively across the mesophyll and may also be organised into bundles. In the leaf, the outer layer of epidermis forms trichomes [62], a specific type of hair, that can be highly distinctive in its shape and surface ornamentation. *I. tinctoria* trichomes are horn-shaped and have surface bumps, making them clearly identifiable and distinguishable from those of other indigo-bearing plants [63]. Such trichomes were observed in the archaeological residues and identified by comparison with those observed on experimentally processed leaves (Fig 4 panels e, f; S8 Fig; S1 Video).

Although highly fragmented (widths ranging between 10–25 μm), the micro-residues that we observed had different morphologies, some appearing rounded or polygonal in section and others elongated with characteristic features (namely structural defects [64]) known as cross markings and dislocations [65–67] (Fig 4a-4c). These features were observed on the archaeological fragments, and on the residues obtained through replicative experiments for the extraction of indigotin from *I. tinctoria* (S7 Fig).

Additional elongated non-coloured micro-residues that show fibrils in transmitted light were also observed. These are likely to originate from different plants, yet to be identified, suggesting that the tools may have also been used to pound plants other than *I. tinctoria*. Since it is plausible that the pebbles are multipurpose tools, micro-remains from other plants are also to be expected.

### Chemo-profiling the micro-residues

The small size of the recovered micro-residues made it impossible to apply conventional molecular techniques (such as HPLC or GC-MS) [68]. Hence, vibrational micro-spectroscopies, notably µ-Raman and µ-FTIR, were considered as the most suitable chemo-profiling techniques to characterise the blue coloured residues and their composition. These methods permitted micrometric-resolved, non-invasive and non-destructive analyses [68].

The blue colour on the fragments was consistently identified as indigotin by μ-Raman analysis, through comparison with two reference substances, a commercial indigotin reference standard (PhytoLab®, reported as Ref1 in Fig 5) and the blue micro-residues obtained from the processing of modern reference *I. tinctoria* (Fig 5d, spectrum Ref 2 and S9 Fig). The spectra of the experimental blue fragments positively matched the 25 archaeological samples investigated, all showing the typical intense µ-Raman peaks of indigotin reference standard at 1575, 545, and 253 cm^-1^ (Fig 5 d and S10 Fig) [68–70].

Indigotin is the main constituent of the indigo dyes and woad that derives from the hydrolysis of the glycoside precursors (primarily Isatan A and Isatan B) in the form of a dark blue crystalline secondary compound. These precursors are glycosidic by-products of the light phase of photosynthesis occurring in the chloroplasts that are stored in the vacuoles of the mesophyll cells of the leaf epidermis. Hence, only these compounds are naturally present in the indigo-bearing plants and they are released from the cells once the leaf is broken down [40,41,44,45]. The chromophore indigotin is not directly available nor visible in the plant during its natural cycle. When the leaf epidermis is pounded, tiny fragments can become entrapped within the stone porosity, in turn the precursors are released and start their transformation through enzymatic hydrolysis, oxidation, and dimerization of two indoxyl molecules (S6 Fig).

The micro-residues are characterised by cellulose (C), pectin (P), and lignin (L) with distinctive Raman signals at 378 and 1098 cm^-1^ (C), 850 cm^-1^ (P) and 1604 cm^-1^ (L) [71–73]. The presence of these biomolecules is reported as a diagnostic composition component for phloem elongated cell walls (including sclerenchyma fibres) [71,72]. Cellulose (C) is the polysaccharide with the highest concentration of plant fibres [71] and its prominent bands at 378 and 1098 cm^-1^ are observed in all the archaeological samples (Fig 6 panel d). Namely, the signal at 378 cm^1^ corresponds to a specific cellulose, that is polymorph I (CI), found in native cellulose fibres [74,75], whereas the peak at 577 cm^-1^ is typical of cellulose polymorph II (CII), differing in anhydro-glucopyranose unit conformations [74,76]. Interestingly, a minor occurrence of polymorph II, along with (CI) signals, has been reported as proof of biodeterioration induced in unprocessed plant fibres by ageing processes [72]. Moreover, the presence of lignin (L) and pectin (P) polymers, which are diagnostic features of both leaf and phloem tissues, were also observed in the µ-Raman spectra of the archaeological residues. These bands are not present in the spectra of cotton seed pod hairs [71,72,77]. The signal at 1604 cm^-1^ is attributed to (L) [71–73], a high-weight polyphenol that accumulates in supporting tissues during plant growth. The low intensity of the (L) peak reflects the low concentration of this polymer in the leaf cells [71]. The presence of the 850 cm^-1^ band, assigned to (P) [73], a heteropolymer formed by monosaccharides and uronic acids linked by ester bonds, is another diagnostic peak for phloem residues. The middle lamellae, which cement together elementary individual cells into bundles in plant phloem, are rich in these polysaccharides [73].

**Fig 7:**
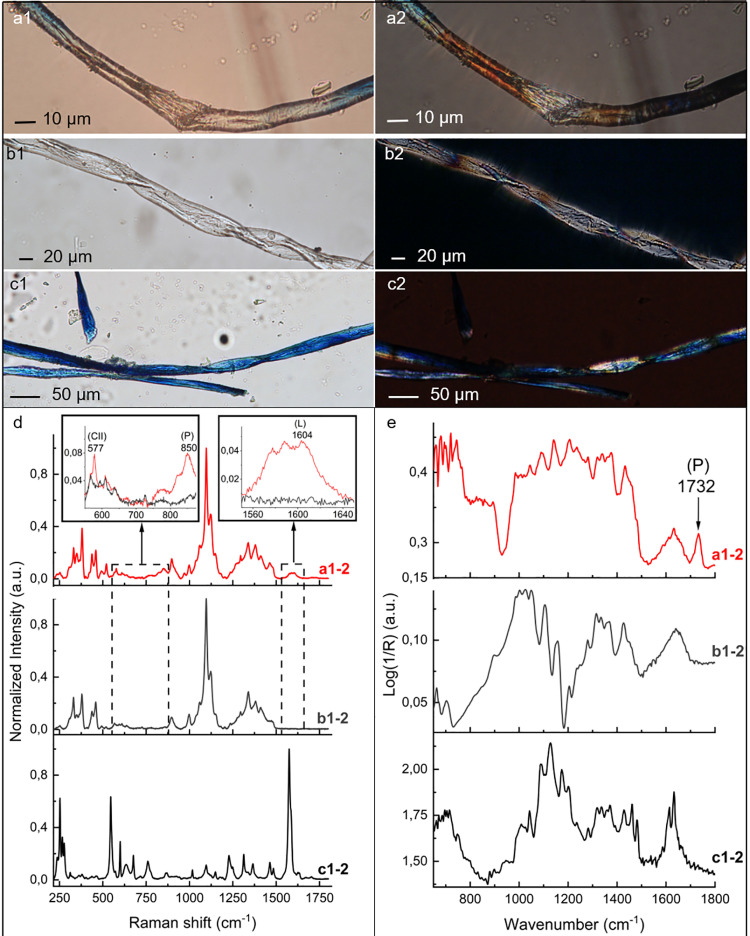
Comparison of blue archaeological micro-residues (a1-a2) with modern non-coloured (b1-b2) and blue (c1-c2) jeans fibres. BF-OM view (a1, b1, c1), P-OM view (a2, b2, c2). Panel d: µ-Raman spectra of a representative archaeological blue micro-residues (Fig 7 d, red line a1-2, reprised from Fig 6 d, line a) compared with blue and non-coloured jeans fibres (Fig 7 d, black c1-2 and grey b1-2 lines, respectively). µ-Raman spectra are presented after normalisation and luminescence background removal. Panel e: reflectance µ-FTIR spectra of the above-mentioned samples. Vibrational bands characteristic of cellulose polymorph II (CII), pectin (P) and lignin (L) are reported. Details of the spectra are reported in S16 Fig.

Our analyses provide distinctive evidence that *I. tinctoria* residues are characterised by detectable signatures of both lignin (L) and pectin (P) along with prominent CI peaks (see spectrum listed as Ref3 in Fig 6 d and S12 Fig). The presence of these bands, along with their relative observed intensities, matches the data obtained from the chemical profiling of the archaeological micro-residues (Figs 6 and 7 b1-2), reflecting a comparable biochemical composition [71,72]. The antiquity of these fragments is confirmed by the additional presence of the cellulose polymorph (CII) (see Fig 6 d spectra a, b and S13 Fig), absent in modern residues, which is coupled with the biodeterioration of the fibres of plant origin [72].

### Testing for blue modern sources of contamination

Natural and synthetic indigotin cannot be chemically differentiated [68,69] (see also S11 Fig). Therefore, the presence of indigotin in the archaeological samples alone cannot exclude the possibility of modern contamination. As a result, we studied both morphological features and chemical signatures to exclude contamination.

*I. tinctoria* is not the only indigo-bearing plant, therefore we considered all the most common ones to check for modern contamination. In particular, we tested *Indigofera tinctoria* L., *Polygonum tinctorium* Aiton, *Isatis indigotica* Fort. and *Baphicacanthus cusia* Bremek, all of tropical origin [36]. Ecological and historical reports make it highly unlikely they were present in the Caucasus under the cool conditions occurring during the Upper Palaeolithic, therefore their occurrence in the archaeological context would indicate modern contamination. The trichome morphology of *I. tinctoria* is highly characteristic and resembles those observed in the GST residues (Fig 4 panels e,f; S8 Fig) [62,78]. Today, indigotin is widely used to colour cloth, notably in the dyeing of blue jeans, that we included in our control samples (Methods, Table 2). This common item of clothing is usually manufactured from cotton (*Gossypium* sp.) [68,79], a seed pod fibre native to tropical and subtropical regions, with numerous species grown commercially today. From an archaeological perspective, cotton is unlikely to be present in the context since the earliest genotyped record of *Gossypium* worldwide is from the site of Qasr Ibrim (Nubia, southern Egypt), Middle Nile valley and dates to around 5500 BC [80]. Cotton seed pod hairs (commonly named cotton fibres) are included in the category of unicellular trichomes; these show a smooth surface without specific ‘ornamentation’ and are ribbon-like, kidney-shaped, and appear flattish in section [81,82]. They vary in diameter and form regular twists or bends along their length, a feature known as convolution [82,83] (see Fig 7 b1-b2, c1-c2; S17 Fig).

Cotton fibres from blue and non-coloured jeans fabric were analysed using both µ-Raman and µ-FTIR spectroscopies. The recorded spectra were compared with the archaeological micro-residues (Fig 7 panels d, e). The cotton fibres sampled from jeans fabrics are composed of at least 90% cellulose, natively present as (CI), as reflected by their vibrational spectra [71,72,77] (grey profiles b1-2 in Fig 7 panels d, e). To the best of our knowledge there are no reports of lignin and pectin signals in the vibrational spectra of cotton fibres [71,72,77].

Furthermore, the µ-FTIR spectrum of the archaeological blue micro-residues exhibit a peak at 1732 cm^-1^, assigned to C=O stretching of ester groups (Fig 7 panel e, red line spectrum a1-2, S15 Fig). The presence of this signal, attributed to pectin [77] (as also confirmed by Raman), matches the spectrum of the experimentally processed modern *I. tinctoria* epidermis fragments (S12 Fig panel b), that is not observed in the jeans cotton sample (Fig 7 panel e, black line c1-2 and grey profile b1-2, respectively). The intensity of this peak is reported to be strengthened by the presence of oxycellulose in aged cellulose cell walls [77]. As far as lignin (L) is concerned, its occurrence cannot be inferred by FTIR analysis due to the presence of the broad and intense water absorption band at approximately 1630 cm^-1^, which overlaps with lignin signals [77] (Fig 7 panel e).

The presence of (P) and (L) in the Dzudzuana micro-residues (Figs 6 panel d and 7 panel d-e red lines a1-2, S13 and S15 Figs) confirms that the residues are not cotton, since these polymers are not present in cotton fibres. All these analyses conducted indicate that the indigotin signal measured on the 25 archaeological blue residues derives from *I. tinctoria*.

## Discussion

Recovering evidence for prehistoric use of plant secondary compounds has barely begun. Although a significant number of plants naturally contain useful secondary metabolites, many require complex processing to access these and make them usable [8]. Even in the case of food, some nutritious plants require extensive leaching, roasting and/or pounding to eliminate toxins, while extraction of useful medicinal secondary compounds demands a deep knowledge of plants since many can be both medicinal and poisonous, with only the correct processing and dosage making the difference [8]. The processing of plants cannot be simply assumed or ignored as it formed part of the complex tapestry of Palaeolithic life. Nonetheless, to demonstrate the use of plants, exhaustive analytical studies are required and studies such as those presented here, is one way to achieve this.

We have presented a multi-analytical procedure for the identification of residues, incorporating both morphological and biomolecular analyses, an approach still quite rare in Palaeolithic research when applied to GST.

By considering the antiquity of the blue micro-residues under study, excluding contamination was given high priority [11,51]. Therefore, we applied a stringent procedure during both the sampling activities in the museum and then during lab-processing. All the control-tests proved negative for the presence of indigotin. Also, modern garments such as blue jeans were tested as possible contaminants and our approach proved crucial to discriminate archaeological blue micro-residues from cotton, a putative source of modern contamination.

The secondary compound identified, indigotin, is not present as such in the indigo-bearing plant and it results from the enzymatic hydrolysis of its precursors, when they are released by pounding the leaves. These are by-products of the photosynthesis; hence they are naturally contained in the cells of the leaves [40–42,44,45]. This first mechanical process is propaedeutic to the following radical cross-coupling of indoxyl, with either isatin (derived from indoxyl oxidation) or a second indoxyl unit, resulting in the formation of indirubin and indigotin chromophores respectively [36,40,44,84,85], in the form of a blue powder insoluble in water and other polar solvents.

Our experiments demonstrated that this part of the *chaîne opératoire* is common to all the indigo-bearing plants, *Indigofera tinctoria* L. (true indigo), *Polygonum tinctorium* Aiton (syn. *Persicaria tinctoria*, Japanese indigo), *Baphycacantus cusia* Bremek (common conehead), *Isatis indigotica* Fort. (Chinese woad), and *Isatis tinctoria* (woad). However, the latter is the only species naturally present across western Eurasia [36]. The paleogenomic signature indicates the presence of *Isatis* sp. at the Palaeolithic site of Aghitu-3 in Armenia (Caucasus) around 36.000 years ago [14], thus making *I. tinctoria* consistent with the source of the identified compound, indigotin, at Dzudzuana. Since the release of these precursors can occur only when the leaves are broken down, the presence of blue fragments found entrapped in the crevices of the Dzudzuana GSTs is unlikely to be accidental.

This study presents an innovative research design, a stringent sampling procedure, and the integrated methodological approach that has led to the identification of indigotin, a secondary compound not naturally present in the plant. In addition, the presence of this molecule provides evidence for the exclusion of contamination. However, there is no direct archaeological evidence for how the *I. tinctoria* plant may have been used. This observation is unsurprising, as plant remains are infrequently preserved in Palaeolithic contexts, making the recovery of direct evidence for the intentional and specific use of plants highly improbable. Indeed, throughout the entirety of the Palaeolithic record, documentation of the deliberate exploitation of particular plant species is exceedingly rare [3–6,14,20,22–25,27,28,86].

As with all archaeological finds, whether they are stone tools or fragments of plants extracted from a GST, their purpose is interpreted based on the context of the finds. *I. tinctoria* leaves are unlikely to have been selected as food because they are extremely bitter and have little nutritional value.

Many plants have extensive medicinal properties including anti-inflammatory, anti-tumour, antimicrobial, antiviral, analgesic, and antioxidant [38,46] and there is extensive evidence for the use of these, not only in human traditional medicine, but also across the animal kingdom. All animals, and even insects, self-medicate [27]. In the case of chimpanzees, they sometimes prepare the plants prior to consumption [87]; Sumatran orang-utans are known to apply to wounds a mashed concoction of *Fibraurea tinctoria* leaves [88,89]. Today, the roots of *I. tinctoria* and other indigo-bearing plants are used in medicine because they contain flavonoids and the leaves contain indigoid-precursor molecules that have preservative, antiseptic, repellent, and protective properties [39,40,45,46]. It is therefore entirely within the behavioural context of humans, from all Palaeolithic periods, to use plants to self-medicate.

*I. tinctoria* is also known as a source of indigotin, a well-established blue chromophore obtained by the oxidation of precursors naturally present in the cells of the leaves. The use of *I. tinctoria* to obtain a blue hue is well known, and this knowledge extends into later prehistory [36,40,90–92]. The use of this plant has been recorded as dye since Egyptian times, the earliest written source being the *Papyrus Graecus Holmensis* (also known as the Stockholm papyrus, retrieved in the XIX century) [93]. However, while the possibility exists that *I. tinctoria* was transformed into woad dye and used during the Early Upper Palaeolithic, there is currently no archaeological evidence for this.

However, and more broadly, colour was a part of Upper Palaeolithic life and the use of mineral pigments is well known, in particular in rock art where red, yellow, white and black are present across the Eurasian continent and the Indonesian archipelago from around 40,000 years ago [94–96]. Blue is a relatively rare colour in nature and to the best of our knowledge, blue pigment (mineral-based) from Palaeolithic contexts has only been reported for Siberian figurines [97].

Plant-based dyes are first reported from the Natufian Epipalaeolithic layers of Kebara cave (~13,500–11,650), where red was extracted from *Rubia tinctoria* roots [92,98]. Seeds of *I. tinctoria* were first recorded in 5000-year-old Neolithic sites in France (e.g. Bouches-du-Rhône) [90,91], but to date, there is no archaeological evidence for the use of *I. tinctoria* prior to the Iron Age.

The multi-analytical approach for the identification of residues, incorporating both morphological and biomolecular procedures is still quite rare in Palaeolithic research while the level of analytical resolution of Upper Palaeolithic GSTs presented here, is yet rarer. The use of plants needs to be demonstrated and exhaustive studies such as those presented here, is one way to achieve this. Use of plants cannot be simply assumed or ignored, even though demonstrating it is time-consuming or complex, as it formed an integral part of the complex tapestry of Palaeolithic life. Our approach, based on the integration of imaging and biomolecular detection, makes the present analysis of a plant comprising both a natural medicine and a dye, the first of its kind for the Upper Palaeolithic.

## Conclusive remarks

This is the first instance where indigotin, a secondary compound derived from plants, has been identified in archaeological micro-residues recovered from stone pebbles dating to the Upper Palaeolithic. Our reproducible analytical approach has provided robust morphological and chemical evidence of the mechanical breakdown of leaves to release indoxyl glycoside precursors—by-products of photosynthesis—that are transformed into indigotin through fermentation. The resulting indigotin is a secondary compound not initially present in the plant.

To the best of our knowledge, our data represents the earliest direct evidence for the processing of a non-nutritional plant, *Isatis tinctoria*, using unmodified pebbles. This discovery offers a rare glimpse into a dimension of Upper Palaeolithic life that extends beyond basic subsistence, further enriching our understanding of the behavioural complexity of *Homo sapiens*. Regardless of the exact purpose for indigotin extraction, the presence of this molecule in a Palaeolithic context is unprecedented, paving the way for future analyses of secondary compound processing from the Palaeolithic period onwards. Whether it was used as a dye, a medicinal agent, or both remains uncertain, providing intriguing avenues for further research.

## Methods

All the techniques and the equipment employed are summarized in Table 1.

### Structural characteristics of the stone pebbles

Synchrotron X-ray computed tomography (SR-μCT), conducted at the SYRMEP beamline of the Elettra Synchrotron facility, Trieste (Italy), was used to reconstruct the internal structure of the stones and its porosity, highlighting the presence of pores that can entrap residues. Three rock samples from the reference material were stacked vertically and investigated with multiple CT scans at different heights, using an ORCA Flash 4.0 Hamamatsu sCMOS detector with a 17 μm thick GGG scintillator screen. For each tomographic scan, 1800 X-ray projections were acquired over a 180° rotation (0.1° angular step) by means of a 2048×2048 pixels detector, with an exposure time of 2000 ms and an effective pixel size of 0.9 μm. The samples were investigated using a polychromatic radiation, pre-filtered with 1.5 mm of Si and 1.0 mm of Al, resulting in a mean beam energy of 27 keV. Edge enhancement by means of propagation-based phase-contrast was obtained using a sample-to-detector distance of 15 cm.

Tomographic reconstructions, including phase retrieval pre-processing, were carried out using the STP (SYRMEP Tomo Project) software [99]. Different values of the ring removal filter and of δ/β ratio for phase retrieval correction were used, depending on the characteristics of the samples. The 32-bit.tiff reconstructed image stacks were then converted to 16-bit in order to process the datasets with available imaging software like Fiji (ImageJ), Dragonfly, CTAn and VGStudioMax.

### Stone pebbles surface microtopography

A variety of microscopy techniques (see Table 1) were employed to inspect the archaeological tools, the reference artefacts, and residues.

The stereomicroscope was used to identify areas characterised by the presence of wear patterns on the moulds. The working distance of this equipment also allowed the direct inspection of the surface of the reference pebbles. Regions identified on moulds as real contact areas were also analysed in more detail with the reflected light OM.

### Scanning confocal microscopy

In order to observe functional patterns at the nanoscale, selected areas of the surface of the moulds were scanned using both white light and UV laser with 20× (NA: 0.40; FN: 26.5; WD: 12 mm), 50× (NA: 0.50; FN: 26.5; WD: 10.6 mm), 100× (NA: 0.80; FN: 26.5; WD: 3.4 mm) objectives. Moreover, the very same technique was used to observe the micro-residues found adhering to the moulds. Subsequently, areas with use-wear traces were scanned using the profiling system operating in confocal mode to acquire microtopographic maps. Also, an unused area was scanned for cross-check. A total of 84 areas on moulds from Dzu S1 (48 areas), Dzu S2 (10 areas), and Dzu S5 (26 areas) were measured. Lenses with different magnifications (10× and 20×) were used to obtain multi-scale information. The 10× lens, with a numerical aperture NA: 0.30, providing a field of view (FOV) of 1270×950 μm, an optical resolution (X/Y) of 0.47 μm, and a vertical resolution of less than 30 nm, was used to scan 80 regions, each covering an area of 850 μm². The 20× lens, with a NA of 0.50, a FOV of 636.61×477.25 μm, an optical resolution (X/Y) of 0.28 μm, and a vertical resolution of less than 15 nm was used to scan 4 regions each covering an area of 420 μm² on Dzu S2, revealing specific micro-features such as parallel striations.

### Micro-residue morphological study

The micro-residues showed structured morphological features that allowed them to be distinguished from amorphous residues, such as glues, resins, tars, etc. The surface of the moulds and the associated structured use-related biogenic residues (SU-RBR) were inspected with both OM - bright light and polarised light - the Confocal LSCM and with SEM equipped with a lanthanum (La) filament as thermionic source. The SEM was used with the aim of inspecting and morphologically characterising the micro residues extracted from both the stone sonication and the moulds as well as the details of the surface topography. For observations with the SEM, coating was necessary (sputter palladium, Pd), and enabled using the 3–5 keV acceleration voltage with a working distance (WD) ranging between 3 and 7 mm. Only a selected portion of the mould was exposed, while the rest was protected and kept uncoated for future analysis. The smallest spot-size beam diameter was used in order to achieve the best spatial resolution, depending on the sample orientation and surface topography. The detector was used in In Lens SE1 or BSE modes. The average size of the leaf epidermis micro-residues is less than 30 μm minimum, up to 500 μm maximum. This strongly constrained the choice of the analytical tools and the methodological approach. It is the main reason why microspectroscopy (µ-Raman and µ-FTIR) was applied for the chemo-profiling rather than the conventional chromatographic analysis (HPLC or GC-MS). The size also restricted further investigation of the anatomical structure of the epidermis fragments (e.g., in cross or longitudinal sections) by means of different microscopes.

### Micro-residues chemo-profiling

μ-Raman and μ-FTIR measurements were always performed before SEM observations since metallic coating of the micro-residues or moulds’ surface interferes with these spectroscopic analyses. The µ-Raman and μ-FTIR were used to characterise both the blue and non-coloured biogenic micro residues, as well as reference materials (for spectra collection parameters see S3 and S4 Tables), since they are non-invasive and non-destructive and guarantee micrometric spatial resolution. Furthermore, vibrational spectroscopies enabled the collection of information on the molecular composition of the leaf epidermis fragments’ matrix in which the dye is adsorbed.

### µ-Raman

Raman spectroscopy is known as a powerful method for the identification of dyes, in particular when the laser exciting light is in resonance or pre-resonance with the electronic transitions of the dyestuff [100]. After initial tests performed with different exciting lines (514, 633 and 785 nm), including an extended spectral range (100–4000 cm^-1^), it was decided that the best conditions to characterise both dye and micro-residues was to excite them at 785 nm, focusing on the most intense vibrational modes between 200 and 1800 cm^-1^. Furthermore, preliminary investigation (live mode by means of oscilloscope) was conducted on at least three different spots of each single micro-residue. Subsequently, the most significant region was selected for longer inspection, ensuring a good signal-to-noise ratio of the spectrum.

The measurements were carried out with a μ-Raman Renishaw InVia spectrometer (see Table 1) and a 4 cm^-1^ spectral resolution was achieved by this setup. The spectrometer was coupled with a Leica DM LM optical microscope. Spectra were collected in a backscattering configuration, through a 50× objective (NA: 0.75) and with power on samples below 150 mW, which prevents fibres matrix degradation; on the other hand, when laser was focused on a blue dyed region, power was kept below 15 mW to prevent indigotin photooxidation.

The signal was calibrated before each acquisition session, relying upon the matching with the internal silicon wafer main band. Data analysis, involving intensity normalisation and luminescence background removal, was performed with the Wire 4.4 software. The obtained spectra were plotted by means of OriginPro 2021 software.

### µ-FTIR

FTIR spectroscopy is a complementary vibrational technique and was used to corroborate and, eventually, add information to the μ-Raman results [101]. Indeed, FTIR does not suffer from photo-induced luminescence emission interference as Raman does. µ-FTIR measurements were performed with a Bruker Lumos II FT-IR microscope, equipped with a LN-MCT (liquid nitrogen cooled - HgCdTe) high performance detector. Spectral resolution was set to 4 cm^-1^. Measurements were carried out in reflectance mode, to ensure a contactless approach, whereas background contribution was collected by the reflected radiation impinging on a gold film. Basic data manipulation (Fourier transform of interferograms) was performed by Opus 8.8 software and the obtained spectra plotted by means of OriginPro 2021 software.

### Modern plant reference collection

#### Isatis tinctoria.

Collecting the leaves of *I. tinctoria* at different time periods was necessary as the content of the precursors and the relative final compound, indigotin, vary across the year [102]. The leaves are richer in compounds during spring, but the content can also vary according to the external climatic conditions (humidity and drought). The plants were sourced in the rosette stage in the Site of Community Importance (SIC) and Special Area of Conservation (ZSC) of Marano di Valpolicella (IT3210002, Verona, IT). The identification was made by LL under the guidance of Daniele Zanini, Scientific Director of the *Orto Botanico di Novezzina* (Ferrara di Monte Baldo, Verona, IT).

Because indigotin is a chromophore obtained from pounded leaves, a series of replicative field experiments were carried out [37,103] at Corte Badin (Verona Italy). Details of the processing procedure can be seen in the video (see S1 Video). In order to perform the replicative experiments, the selected stone pebble was cleaned in the lab by soaking it in a beaker for 30 minutes in different solutions: HCl (5%) and, H₂O₂ (5%). Each treatment was followed by sonication in ultrapure water for 30 minutes. Moulds of the working surfaces were then taken at time zero (T₀). Experiments were carried out at different times of the year according to the *I. tinctoria* life cycle [37,102]: in early spring (before flowering), summer, and early autumn. 800 g of leaves, cut from the first year (rosette stage), were immediately processed using the stone pebble in a wooden mortar for approximately 30–40 minutes. The pestle was reused in the subsequent replicative experiments, without being washed, and stored in a sealed bag until the next use. The mechanical processing was characterised by a combination of vertical action (such as pounding) and horizontal linear motion that shredded the leaves, with these actions repeated until a poultice was formed. All steps of the process were photographed and video-recorded (S1 File; S1 Video). Our experiments have shown that the poultice tends to overflow from the direct contact area and adhere to the hand operating the stone, meaning the residues can easily spread across multiple areas, not necessarily limited to those in direct contact with the working area (S1 Video). Fragments of the leaves remained trapped in the uneven surface topography of the stone.

### Indigo-bearing plants

To exclude the presence of other indigo-bearing plants, we tested the most common ones [36], and in particular *Indigofera tinctoria* L., *Polygonum tinctorium* Aiton*, Isatis indigotica* Fort. and *Baphicacanthus cusia* Bremek. The plants were sourced from a Chinese Pharmacy shop in Hangzhou, China, and were collected from regions in subtropical to tropical climates namely Zhejiang Province, Hunan Province, Anui Province, and Guangdong Province respectively.

All the samples underwent the same processing under a fume hood: pounding the leaves, adding a few mL of ultrapure water and letting the fermentation process start. Little balls were made and left to dry for several weeks. The balls were ground and the blue pellet was observed for morphological features and analysed with µ-Raman.

#### Jeans fibres (*Gossypium* sp.).

Indigotin is widely used to colour textiles, in particular in the dyeing of blue jeans, a common item of clothing usually manufactured from cotton [68], a seed pod hair. In particular, upland cotton (*Gossypium hirsutum* [79,81,82]) accounts for 90% of modern production [104]. Thus, to ensure that the presence of archaeological micro-residues is not the result of modern contamination, cotton fibres from blue and non-coloured jeans fabric were sourced. Morphological and vibrational analyses performed on these fibres were compared to the archaeological micro-residues.

## Supporting information

S1 Table Information regarding the stone pebbles and the sampling in the museum. The stones were dug during the 2004–2007 field works. The strata have been excavated by artificial cuts of 5 or 10 cm.
(DOCX)

S2 Table Summary of the moulds analysed and of the residues observed. (DOCX)

S3 Table Chemo-profiled sample description and µ-Raman collection parameters of reported spectra, excited at 785 nm. (DOCX)

S4 Table Chemo-profiled sample description and µ-FTIR collection parameters of reported spectra. (DOCX)

S1 Fig Dzudzuana stone pebble macrographs. Stone pebbles retrieved from the 2002–2007 excavation at Dzudzuana cave, Layer D, squares G7, G8 and I18. All stones except for Dzu S4 are considered in this study.
(FIG)

S2 Fig Moulds preliminary inspection. (a) An example of the areas targeted by the moulding technique and location of the mould on the pebble, (b) example of the peel-off effect extracting soil entrapped in the crevices, (c) blue and white fibres extracted when observed under transmitted light OM: (d) Dino-Lite digital microscope: use-wear traces on Dzu S1 mould 2.
(FIG)

S3 Fig Moulds use-wear analysis. (a-d) use-wear traces observed on Dzu S1 m2 by means of laser. scanning confocal microscopy (LSCM): polish and striations seen with white light (a-b) and UV laser (c-d) (scale bar 100 µm). SEM (e-f): smooth and flattened areas as seen on Dzu S2.
(FIG)

S4 Fig Micro residue extraction from moulds. Different techniques used to analyse soil still adhering to the mould, as reported in Methods, to examine for the presence of residues and use-wear traces. Selected moulds were subsampled and only a portion was considered in order to maintain the other part for future analyses. (a): mould indentation (cut portion) presenting soil was observed with OM, SEM, and LSCM. The remaining portion was studied with OM and confocal profilometer; (b): scraping of soil from the mould’s surface and dilution in ultrapure water. Residues observed with OM, LSCM and spectroscopic techniques; (c): sonication of mould section in ultrapure water, residues observed in the same manner as (b).
(FIG)

S5 Fig Blue archaeological residues micrographs. Imaging of blue archaeological residues by optical microscopy in bright field (a, c) and polarised light (b, d), by scanning confocal microscopy in UV laser (e), and white light (f) (scale bar 20 µm). White arrows indicate kink-bands/dislocations along the bast fibres.
(FIG)

S6 Fig Non-coloured archaeological fragments micrographs. Imaging of non-coloured archaeological residues by optical microscopy in bright field (a, c) and polarised light (b, d), by scanning confocal microscopy UV (e), and white lights (f) (scale bar 20 µm).
(FIG)

S7 Fig *Isatis tinctoria* fibres micrographs. Imaging of blue and non-coloured fibres from replicative experiments on *I. tinctoria* by OM bright field (a-c) and polarised light (d-f). White arrows indicate kink-bands/dislocations along the length of the fibres.
(FIG)

S8 Fig Archaeological trichomes micrographs compared with *I. tinctoria* ones. Imaging of trichomes (plant hairs): note the characteristic bumps on *I. tinctoria* trichomes. (a, b) archaeological micro-residues; (c, d) modern *I. tinctoria* leaves. The micrographs were acquired with bright field OM (a, c) and SEM (b, d).
(FIG)

S9 Fig µ-Raman spectra of blue micro fibres extracted from *I. tinctoria.* Modern reference: Normalised Raman spectra of different blue micro fibres (a, b, c) extracted from *I. tinctoria* leaves, excited at 785 nm and after normalisation and luminescence background removal. Characteristic bands of indigotin (Ind) and cellulose (C, CI) are indicated by their Raman shift.
(FIG)

S10 Fig µ-Raman spectra of blue archaeological micro residues. Archaeological fragments: Normalised Raman spectra of blue micro residues (a: S1 m3; b: S2 m6; c: S6 m2), excited at 785 nm and after luminescence background removal. Characteristic bands of indigotin (Ind) and cellulose (C, CI) are indicated by their Raman shift.
(FIG)

S11 Fig µ-Raman spectra comparison between natural woad and synthetic indigotin. Normalised Raman spectra of natural woad dye extracted from modern *I. tinctoria* leaves according to hot-water extraction (see S1 File), compared with synthetic indigotin standard. Both were excited at 785 nm and they are shown after luminescence background removal.
(FIG)

S12 Fig µ-Raman and µ-FTIR spectra of non-coloured micro fibres extracted from *I. tinctoria.* Normalised Raman after luminescence background removal (panel a), and reflectance FTIR (panel b) spectra of non-coloured micro fibres collected from modern *I. tinctoria* leaves. Characteristic bands of cellulose (C), including its polymorph I (CI), pectin (P) and lignin (L) are indicated by their Raman shift and wavenumber.
(FIG)

S13 Fig µ-Raman spectra of non-coloured archaeological micro residues Normalised Raman spectra of archaeological non-coloured micro fragments from Dzu S6 m3 (a) and Dzu S1 m7 (b), reprised from Fig 6, excited at 785 nm and after luminescence background removal. Characteristic bands of cellulose (C), including its polymorphs I (CI) and II (CII), pectin (P) and lignin (L) are indicated by their Raman shift.
(FIG)

S14 Fig µ-Raman spectra of archaeological micro residues (reprised from Fig 6 panel d). µ-Raman spectra of blue and non-coloured archaeological fragments. (a): spectrum of a non-coloured region of a blue residue; spectra b and c of non-coloured residues; spectra Ref3 of non-coloured *I. tinctoria* fibre obtained from the processing of modern leaves (reported as reference). (b and c): zoomed region of interest of the spectra reported in (a). This figure supports Fig 6 d of the main text.
(FIG)

S15 Fig Reflectance µ-FTIR spectra of blue archaeological micro residues. Reflectance micro-FTIR spectra of archaeological blue fragments from Dzu S1 m3 (a) and Dzu S2 m6 (b). Characteristic bands of cellulose (C) and pectin (P) are indicated by their wavenumber.
(FIG)

S16 Fig µ-Raman spectra of blue archaeological residues compared with jeans fibres one (reprised from Fig 7 panel d). µ-Raman spectra of blue and modern fibres. (a): spectrum a1-a2 of blue archaeological residues, while spectrum b1-b2 is a modern non-coloured jeans fibres reported for comparison. (b and c): zoomed region of interest of the spectra reported in (a). This figure supports Fig 7 d of the main text.
(FIG)

S17 Fig Jeans fibres micrographs. Blue jeans fabrics (*Gossypium* sp.) used for cross-reference to verify any potential modern contamination. (a) blue jeans fabric and detail of a thread under the stereomicroscope; (b) blue jeans fibres under OM, in the insert characteristic features of cotton are visible: ribbon-like kidney-shaped fibres, flattish in section that vary in diameter and form twists or bends along their length, a feature known as convolution; (c-d) the same thread as in (a and b), observed with SEM. Insert in (d) shows the structure of the cotton fibre.
(FIG)

S1 File Replicative experiments, *Isatis tinctoria* L. processing and woad extraction. (PDF)

S1 Video Replicative experiments, *Isatis tinctoria* L. processing and woad extraction. (MP4)
