# Correction: Direct evidence for processing Isatis tinctoria L., a non-nutritional plant, 32–34,000 years ago

**DOI:** 10.1371/journal.pone.0335850

**Published:** 2025-10-30

**Authors:** Laura Longo, Mauro Veronese, Clarissa Cagnato, Giusi Sorrentino, Ana Tetruashvili, Anna Belfer-Cohen, Nino Jakeli, Tengiz Meshveliani, Moreno Meneghetti, Alfonso Zoleo, Antonio Marcomini, Gilberto Artioli, Elena Badetti, Karen Hardy

The ORCID iDs are missing for the second, fourth, fifth, sixth, seventh, nineth, tenth, eleventh and fourteenth author. Please see the authors’ respective ORCID iDs here:

- Author Mauro Veronese’s ORCID iD is: https://orcid.org/0009-0000-1402-6250
- Author Giusi’s Sorrentino ORCID iD is: https://orcid.org/0000-0001-6500-7439
- Author Ana Tetruashvili’s ORCID iD is: https://orcid.org/0000-0001-9124-8869
- Author Anna Belfer-Cohen’s ORCID iD is: https://orcid.org/0000-0003-3072-6657
- Author Nino Jakeli’s ORCID iD is: https://orcid.org/0009-0000-9094-3683
- Author Moreno Meneghetti’s ORCID iD is: https://orcid.org/0000-0003-3355-4811
- Author Alfonso Zoleo’s ORCID iD is: https://orcid.org/0000-0001-9432-0723
- Author Karen Hardy’s ORCID iD is: https://orcid.org/0000-0003-1127-2397
