# Supplementary material for: Direct evidence for processing Isatis tinctoria L., a non-nutritional plant, 32–34,000 years ago
Source: PLoS One. 2025 May 9;20(5):e0321262. doi: 10.1371/journal.pone.0321262 (PMC12063890; doi:10.1371/journal.pone.0321262)
Supplement: S1 Table — The stones were dug during the 2004–2007 field works. The strata have been excavated by artificial cuts of 5 or 10 cm. (DOCX) [file pone.0321262.s001.docx]

| **Item**  **Identification**  **Provenance** | **Dimensions:**  **Length (L)**  **Width (Wd)**  **Thickness (T)**  **Weight (W)** | **Object biography/**  **Taphonomic history** | **Residue Dislodgement /**  **Extraction** | |
| --- | --- | --- | --- | --- |
| Dzu S1  Sq.  G8c  H: 645-650  Layer D | L = 11.9 cm  Wd = 10.1 cm  T = 3.5 cm  W = 0.675 g | Rinsed in the river  Sediment still adhering  to the surface  Integrity: broken,  part of a pebble | Sonication  at GNM  (Palynology Lab)  Moulding | 2^nd^ moulds  sub-sampling  Sonication of  selected part  of the mould (see Supplementary Fig.4) |
| Dzu S2  Sq.  G8b  H: 645-650  Layer D | L = 14.5 cm  Wd = 11 cm  T = 3.9-5.5 cm  W = 1.376 g | Rinsed in the river  Sediment still adhering  to the surface  Integrity: broken,  part of a pebble | Sonication  at GNM  (Palynology Lab)  Moulding | 2^nd^ moulds  sub-sampling  Sonication of  selected part  of the mould |
| Dzu S3  Sq. G7a  H: 675-680  Layer D | L = 7.9 cm  Wd = 4.8 cm  T = 1.6 cm  W = 0.099 g | Rinsed in the river  Sediment still adhering  to the surface  Integrity: complete oval pebble | Sonication  at GNM  (Palynology Lab)  Moulding | 2^nd^ moulds  sub-sampling  Sonication of  selected  moulds |
| Dzu S4  Sq. I18a+c  H: 350-360  Layer C5  (in contact with Layer D) | L = 8.8 cm  Wd = 1.6 cm  T = 1.4 cm  W = 0.035 g | Rinsed in the river  Integrity: complete  Elongated artifact presenting  areas with evident polishing,  probably a pebble | Sonication  at GNM  (Palynology Lab)  Moulding | 2^nd^ moulds  sub-sampling  Sonication of  selected  moulds |
| Dzu S5  Sq. I8 b+d  H: 440-450  Layer D | L = 9.6 cm  Wd = 6.3 cm  T = 1.3 cm  W = 0.110 g | Rinsed in the river  Integrity: Broken artifact  The central area is concave, probably part of a pebble  (used also as a boiling stone) | Sonication  at GNM  (Palynology Lab)  Moulding | 2^nd^ moulds  sub-sampling  Sonication of  selected  moulds |
| Dzu S6  Sq. I8a  H: 655-660  Layer D | L = 8.6 cm  Wd = 2.6 cm  T = 1.9 cm  W = 0.073 g | Rinsed in the river  Integrity: Broken stone | Sonication  at GNM  (Palynology Lab)  Moulding | 2^nd^ moulds  sub-sampling  Sonication of  selected  moulds |
