# Supplementary material for: Direct evidence for processing Isatis tinctoria L., a non-nutritional plant, 32–34,000 years ago
Source: PLoS One. 2025 May 9;20(5):e0321262. doi: 10.1371/journal.pone.0321262 (PMC12063890; doi:10.1371/journal.pone.0321262)
Supplement: S2 Table — (DOCX) [file pone.0321262.s002.docx]

| **Stone pebbles** | **m1** | **m2** | **m3** | **m4** | **m5** | **m6** | **m7** |
| --- | --- | --- | --- | --- | --- | --- | --- |
| Dzu S1  7 moulds | Soil present  Blue residues retrieved in the soil adhering to the mould | Soil present  Blue residues retrieved in the soil adhering to the mould | Blue residues | Blue residues | Soil present  Blue residues retrieved in the soil adhering to the mould | Blue residues | Blue residues  Non-coloured residues |
| Dzu S2  7 moulds | Soil present  Blue residues retrieved in the soil adhering to the mould | Soil present  Blue residues retrieved in the soil adhering to the mould | Blue residues | Blue residues | Soil present  Blue residues retrieved in the soil adhering to the mould | Blue residues | Blue residues  Non-coloured fragments |
| Dzu S3  4 moulds | Soil present  Blue residues retrieved in the soil adhering to the mould | Soil present  Blue residues retrieved in the soil adhering to the mould | Blue residues | Blue residues |  |  |  |
| Dzu S4  not examined |  |  |  |  |  |  |  |
| Dzu S5  3 moulds | Blue residues  Non-coloured fragments | Soil present  Blue residues retrieved in the soil adhering to the mould | Blue fragments |  |  |  |  |
| Dzu S6  3 moulds | Soil present  Blue residues retrieved in the soil adhering to the mould | Soil present  Blue residues retrieved in the soil adhering to the mould  Non-coloured fragments | Blue fragments  Non-coloured residues |  |  |  |  |
