# Supplementary material for: Direct evidence for processing Isatis tinctoria L., a non-nutritional plant, 32–34,000 years ago
Source: PLoS One. 2025 May 9;20(5):e0321262. doi: 10.1371/journal.pone.0321262 (PMC12063890; doi:10.1371/journal.pone.0321262)
Supplement: S3 Table — (DOCX) [file pone.0321262.s003.docx]

| **Spectrum label**  **(Figure number)** | **Sample and provenience** | **Laser power [mW]** | **Integration time [s]** | **Number of scans** | **Spectral resolution [cm^-1]^** | **Focusing objective** |
| --- | --- | --- | --- | --- | --- | --- |
| a1-a2  (Fig 5) | Blue micro residue from Dzu S1 m7 | 15 | 15 | 30 | 4 | 50× |
| b1-2  (Fig 5) | Blue micro residue from Dzu S6 m2 | 15 | 15 | 30 | 4 | 50× |
| c1-2  (Fig 5) | Blue micro residue from Dzu S5 m3 | 15 | 15 | 30 | 4 | 50× |
| a  (S9 Fig) | Blue micro residue from Dzu S1 m3 | 15 | 20 | 50 | 4 | 50× |
| b  (S9 Fig) | Blue micro residue from Dzu S2 m6 | 15 | 20 | 20 | 4 | 50× |
| c  (S9 Fig) | Blue micro residue from Dzu S6 m2 | 3 | 20 | 20 | 4 | 50× |
| Ref2  (Fig 5) | Blue micro residue from modern replicative *Isatis tinctoria* processing (*Pounding leaves for woad ball preparation*, see S1 File) | 15 | 15 | 20 | 4 | 50× |
| a  (S8 Fig) | Blue micro residue from modern replicative *Isatis tinctoria* processing (*Pounding leaves for woad ball preparation*, see S1 File) | 15 | 15 | 20 | 4 | 50× |
| b  (S8 Fig) | Blue micro residue from modern replicative *Isatis tinctoria* processing (*Pounding leaves for woad ball preparation*, see S1 File) | 15 | 15 | 20 | 4 | 50× |
| c  (S8 Fig) | Blue micro residue from modern replicative *Isatis tinctoria* processing (*Pounding leaves for woad ball preparation*, see S1 File) | 3 | 20 | 20 | 4 | 50× |
| Woad  (S11 Fig) | Experimental woad dye (*hot-water extraction*, see S1 File) | 15 | 20 | 50 | 4 | 50× |
| Ref1 (Fig 5), Indigotin (S10 Fig) | Commercial indigotin reference standard (PhytoLab® GmbH) | 3 | 20 | 25 | 4 | 50× |
| a (Fig 6),  a1-2 (Fig 7 d) | Inhomogeneous blue micro residue from Dzu S5 m3 | 150 | 20 | 20 | 4 | 50× |
| b (Fig 6),  a (S13 Fig) | Non-coloured micro residue from Dzu S6 m3 | 150 | 20 | 20 | 4 | 50× |
| c  (Fig 6) | Non-coloured micro residue from Dzu S5 m3 | 150 | 20 | 15 | 4 | 50× |
| b  (S13 Fig) | Non-coloured micro residue from Dzu S1 m7 | 30 | 20 | 15 | 4 | 50× |
| Ref3 (Fig 6),  a (S11 Fig panel a) | Non-coloured micro residue from modern replicative *Isatis tinctoria* processing (*Pounding leaves for woad ball preparation*, see S1 File) | 150 | 20 | 25 | 4 | 50× |
| b  (S11 Fig panel a) | Non-coloured micro residue from modern replicative *Isatis tinctoria* processing (*Pounding leaves for woad ball preparation*, see S1 File) | 150 | 20 | 10 | 4 | 50× |
| b1-2  (Fig 7 d) | Modern non-coloured cotton fibre (non-coloured jeans) | 300 | 20 | 20 | 4 | 50× |
| c1-2  (Fig 7 d) | Modern indigotin-dyed cotton fibre (blue jeans) | 30 | 20 | 25 | 4 | 50× |
