# Supplementary material for: Direct evidence for processing Isatis tinctoria L., a non-nutritional plant, 32–34,000 years ago
Source: PLoS One. 2025 May 9;20(5):e0321262. doi: 10.1371/journal.pone.0321262 (PMC12063890; doi:10.1371/journal.pone.0321262)
Supplement: S4 Table — (DOCX) [file pone.0321262.s004.docx]

| **Spectrum label**  **(Figure number)** | **Sample and provenience** | **Number of scans** | **Spectral resolution [cm^-1]^** |
| --- | --- | --- | --- |
| a1-2  (Fig 7 panel e) | Inhomogeneous blue micro residue from Dzu S5 m3 | 256 | 4 |
| a  (S12 Fig) | Blue micro residue from Dzu S1 m7 | 256 | 4 |
| b  (S12 Fig) | Blue micro residue from Dzu S5 m3 | 256 | 4 |
| a  (S11 Fig) | Non-coloured micro residue from modern replicative *I. tinctoria* processing (*Pounding leaves for woad ball preparation*, see S1 File) | 256 | 4 |
| b  (S11 Fig) | Non-coloured micro residue from modern replicative *I. tinctoria* processing (*Pounding leaves for woad ball preparation*, see S1 File) | 256 | 4 |
| b1-2  (Fig 7 panel e) | Modern non-coloured cotton fibre (non-coloured jeans) | 64 | 4 |
| c1-2  (Fig 7 panel e) | Modern indigotin-dyed cotton fibre (blue jeans) | 64 | 4 |
