# Supplementary figures and images for: Direct evidence for processing Isatis tinctoria L., a non-nutritional plant, 32–34,000 years ago
Source: PLoS One. 2025 May 9;20(5):e0321262. doi: 10.1371/journal.pone.0321262 (PMC12063890; doi:10.1371/journal.pone.0321262)

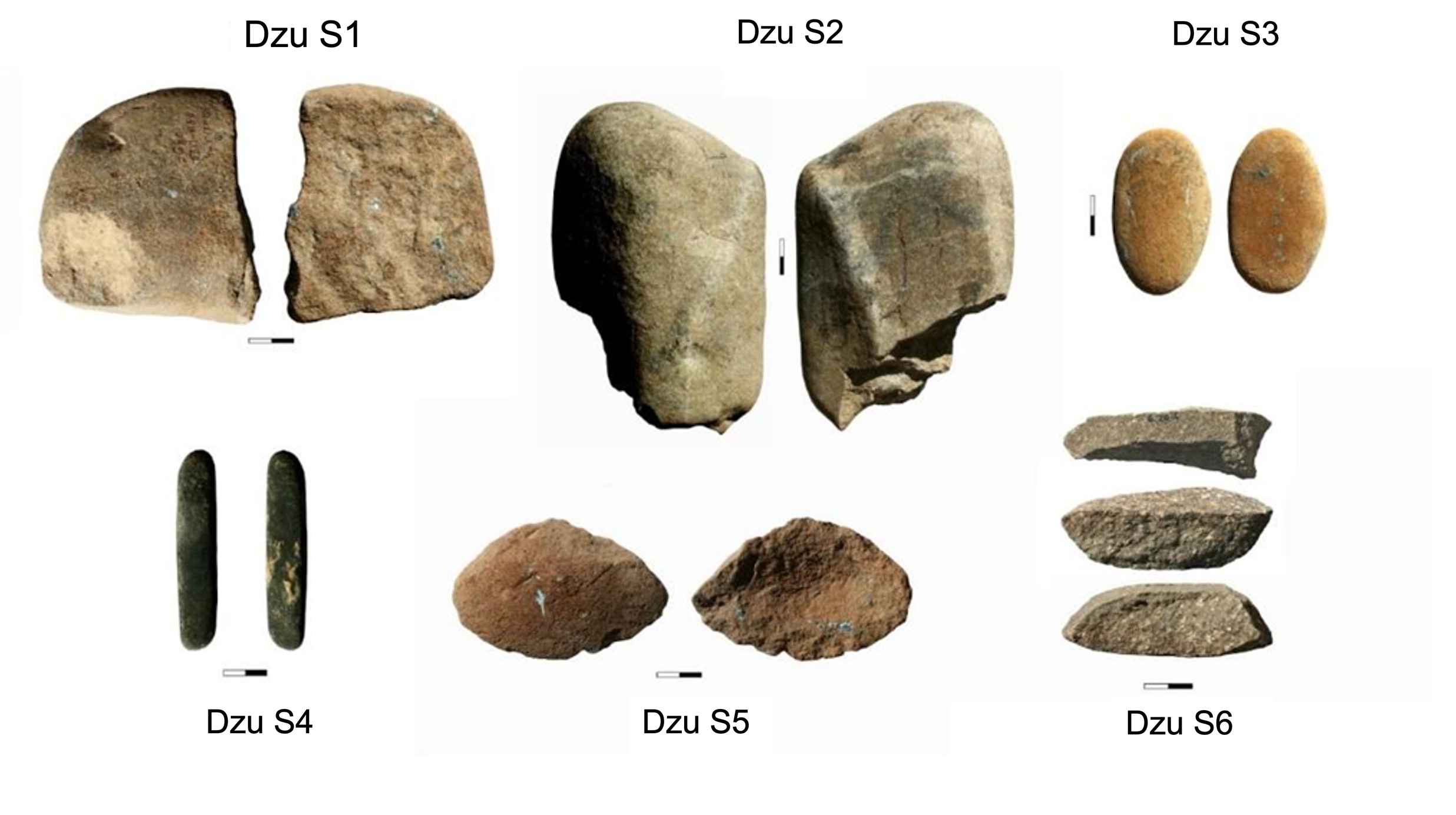

Supplement: S1 Fig — Stone pebbles retrieved from the 2002–2007 excavation at Dzudzuana cave, Layer D, squares G7, G8 and I18. All stones except for Dzu S4 are considered in this study. (FIG) [file pone.0321262.s005.tif]

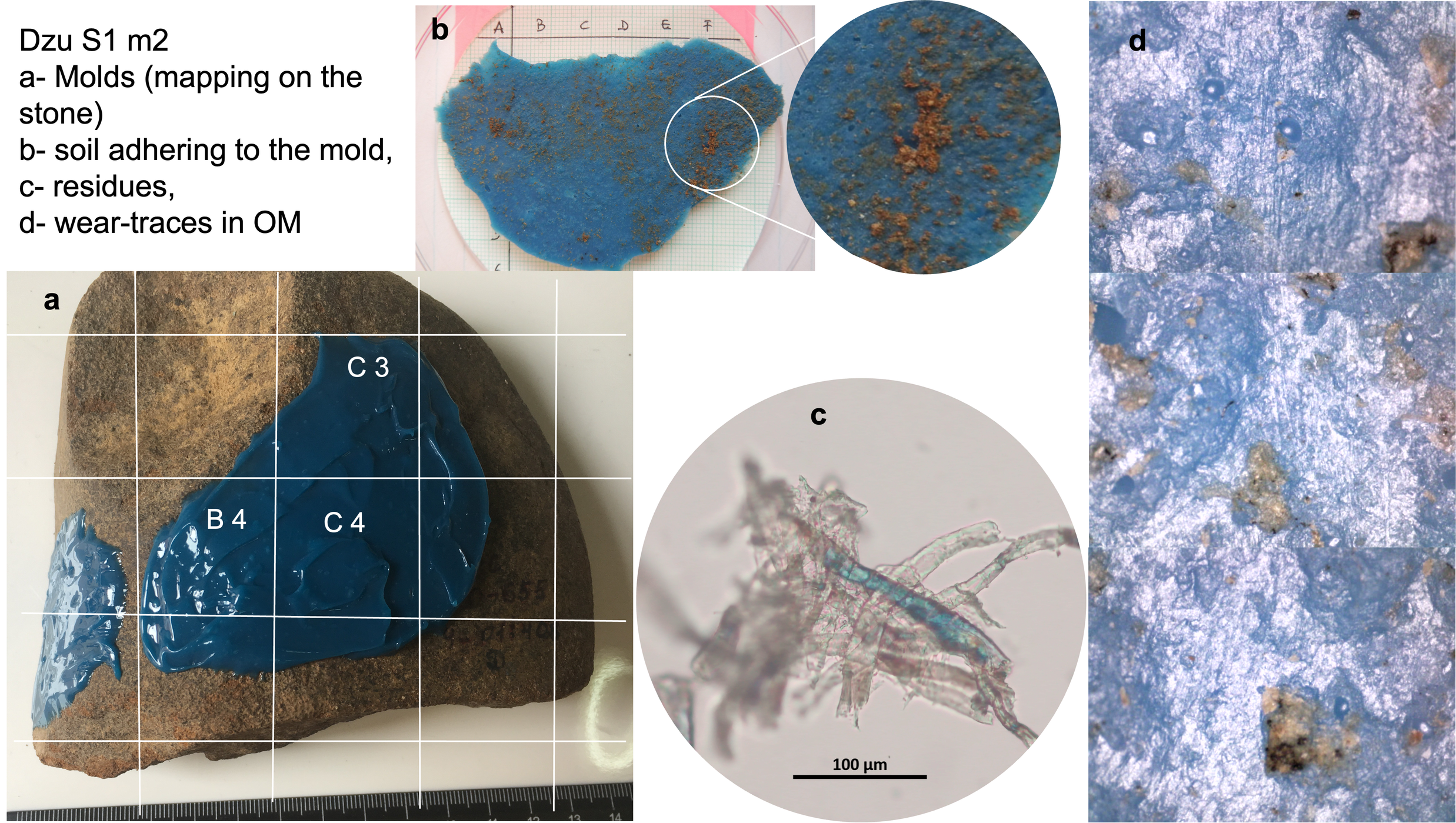

Supplement: S2 Fig — (a) An example of the areas targeted by the moulding technique and location of the mould on the pebble, (b) example of the peel-off effect extracting soil entrapped in the crevices, (c) blue and white fibres extracted when observed under transmitted light OM: (d) Dino-Lite digital microscope: use-wear traces on Dzu S1 mould 2. (FIG) [file pone.0321262.s006.tif]

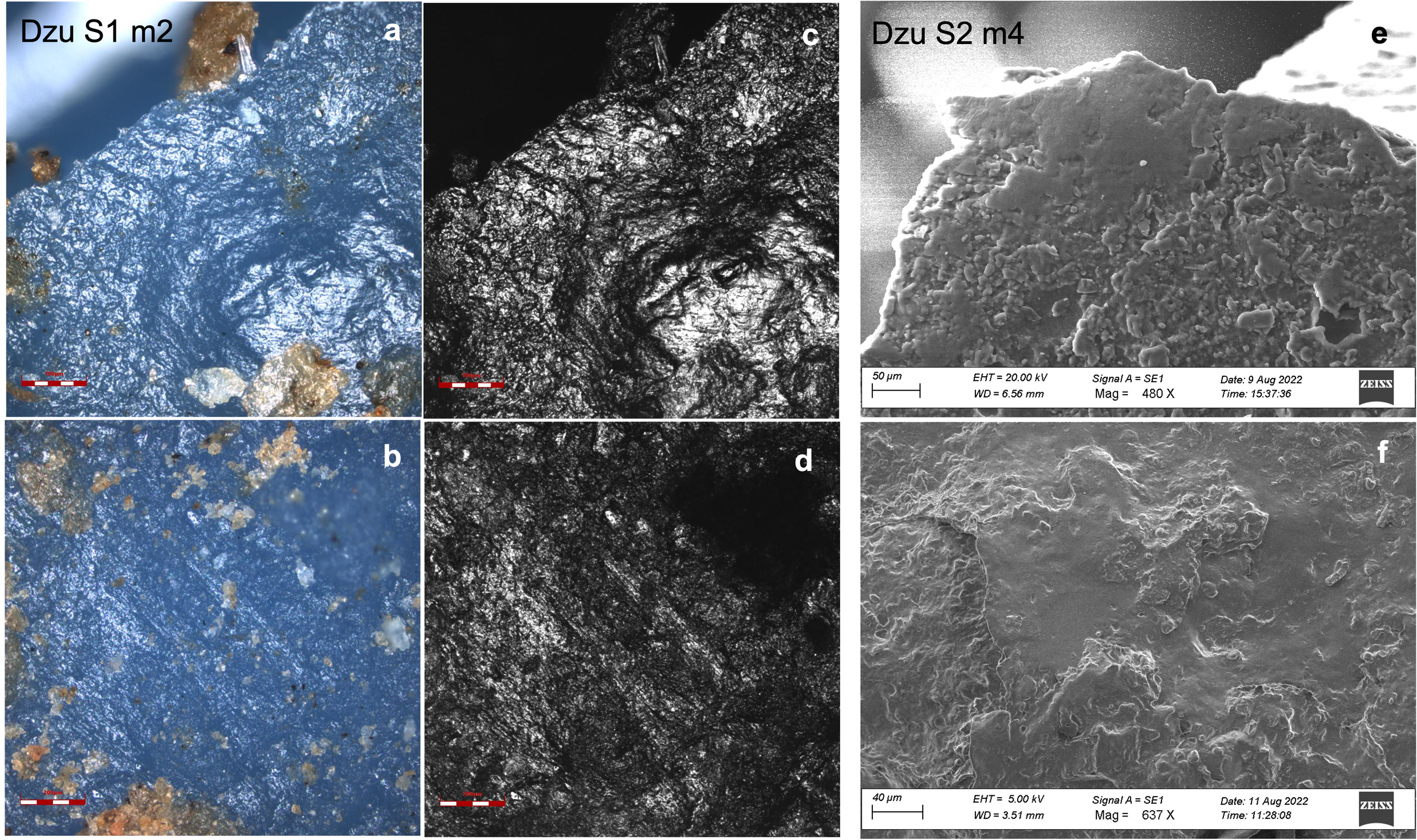

Supplement: S3 Fig — (a-d) use-wear traces observed on Dzu S1 m2 by means of laser. scanning confocal microscopy (LSCM): polish and striations seen with white light (a-b) and UV laser (c-d) (scale bar 100 µm). SEM (e-f): smooth and flattened areas as seen on Dzu S2. (FIG) [file pone.0321262.s007.tif]

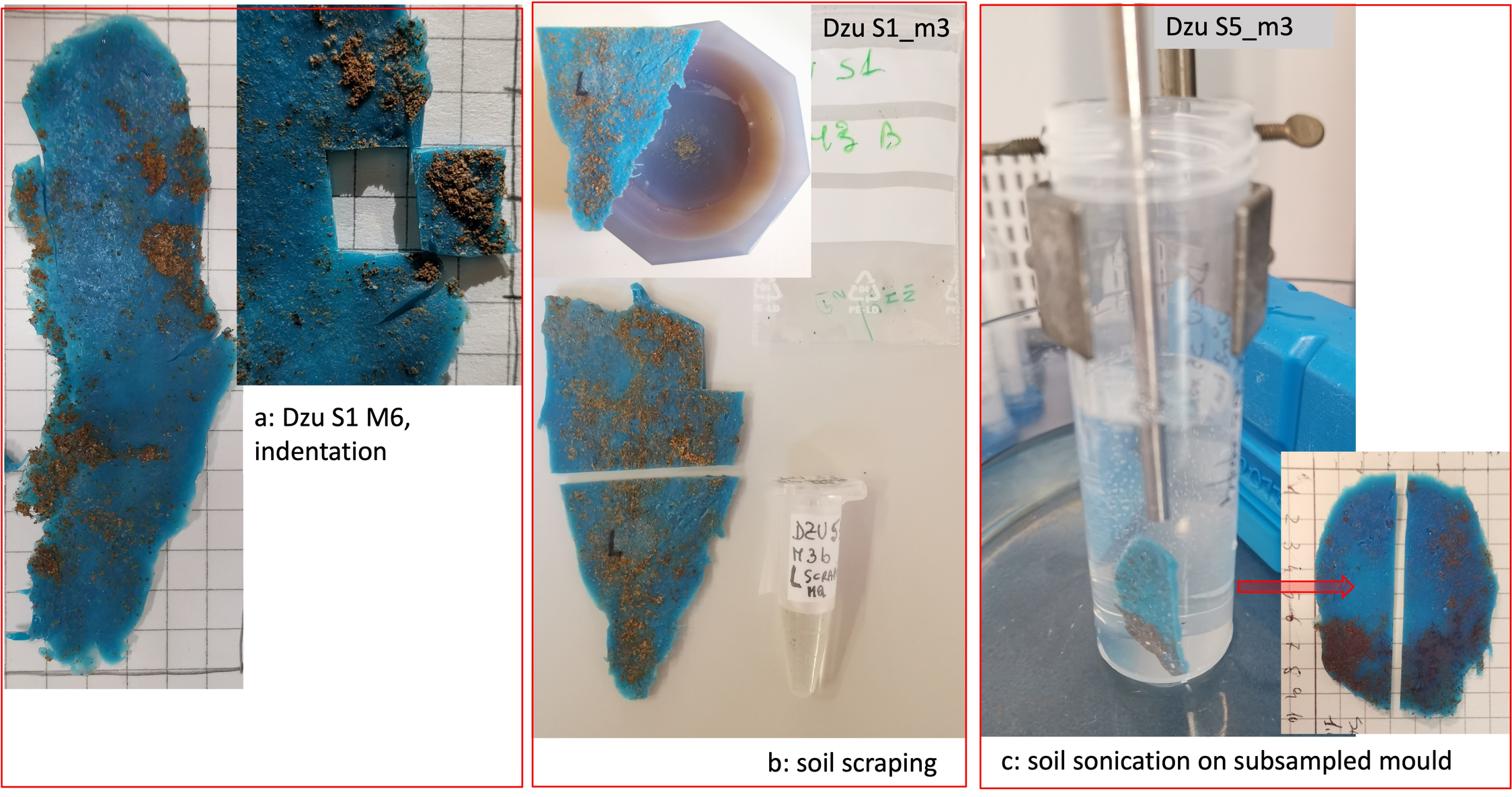

Supplement: S4 Fig — Different techniques used to analyse soil still adhering to the mould, as reported in Methods, to examine for the presence of residues and use-wear traces. Selected moulds were subsampled and only a portion was considered in order to maintain the other part for future analyses. (a): mould indentation (cut portion) presenting soil was observed with OM, SEM, and LSCM. The remaining portion was studied with OM and confocal profilometer; (b): scraping of soil from the mould’s surface and dilution in ultrapure water. Residues observed with OM, LSCM and spectroscopic techniques; (c): sonication of mould section in ultrapure water, residues observed in the same manner as (b). (FIG) [file pone.0321262.s008.tif]

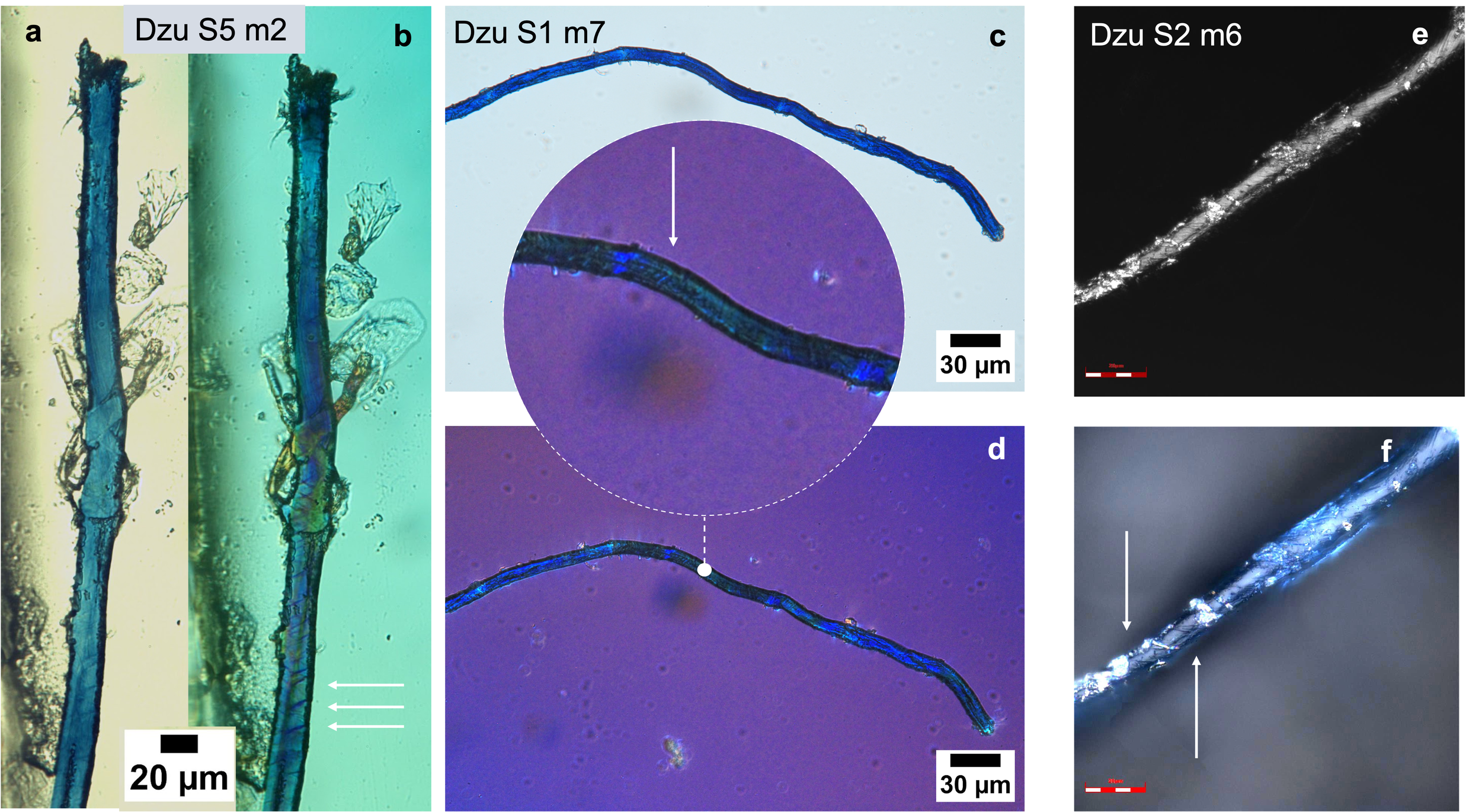

Supplement: S5 Fig — Imaging of blue archaeological residues by optical microscopy in bright field (a, c) and polarised light (b, d), by scanning confocal microscopy in UV laser (e), and white light (f) (scale bar 20 µm). White arrows indicate kink-bands/dislocations along the bast fibres. (FIG) [file pone.0321262.s009.tif]

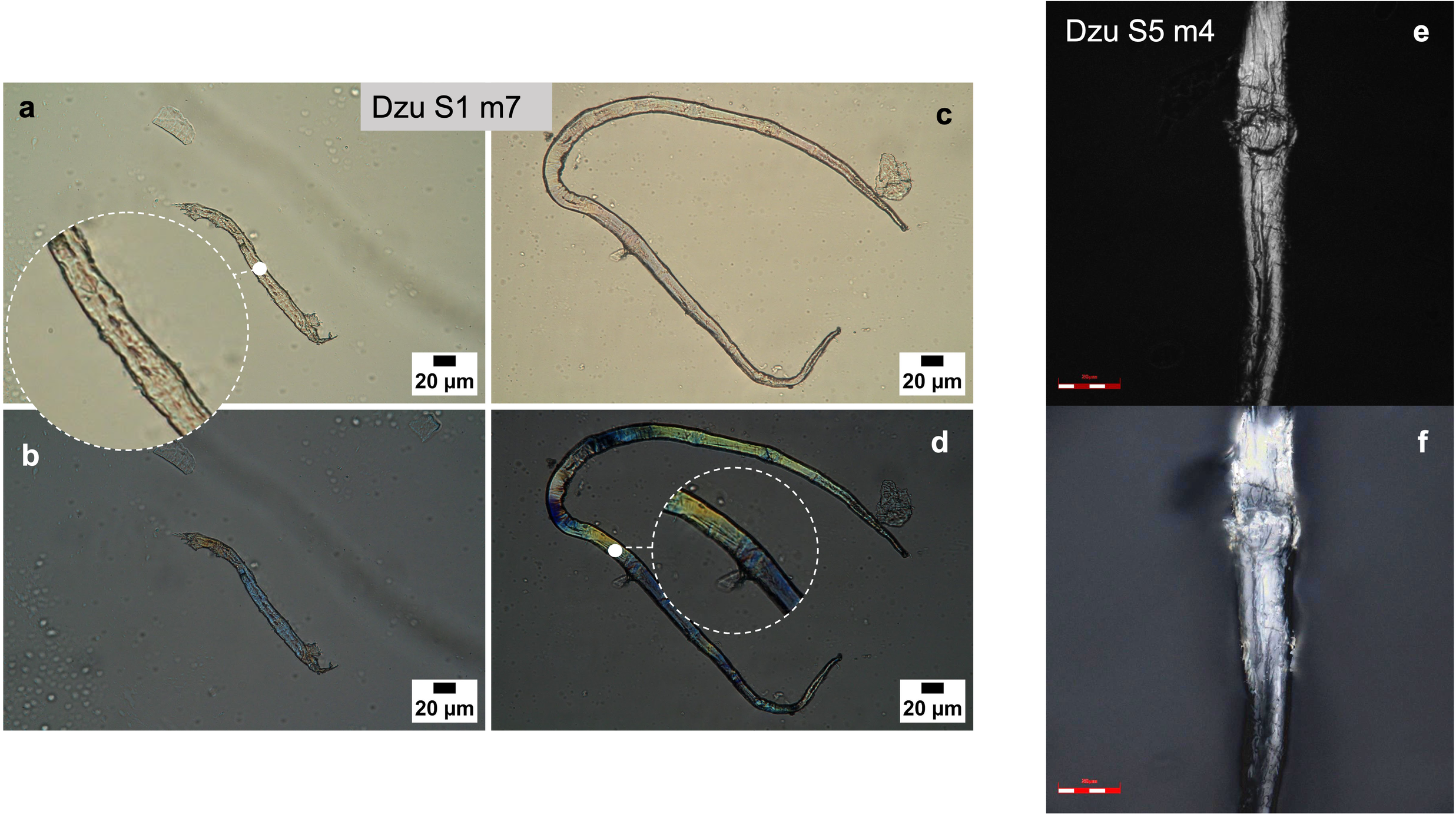

Supplement: S6 Fig — Imaging of non-coloured archaeological residues by optical microscopy in bright field (a, c) and polarised light (b, d), by scanning confocal microscopy UV (e), and white lights (f) (scale bar 20 µm). (FIG) [file pone.0321262.s010.tif]

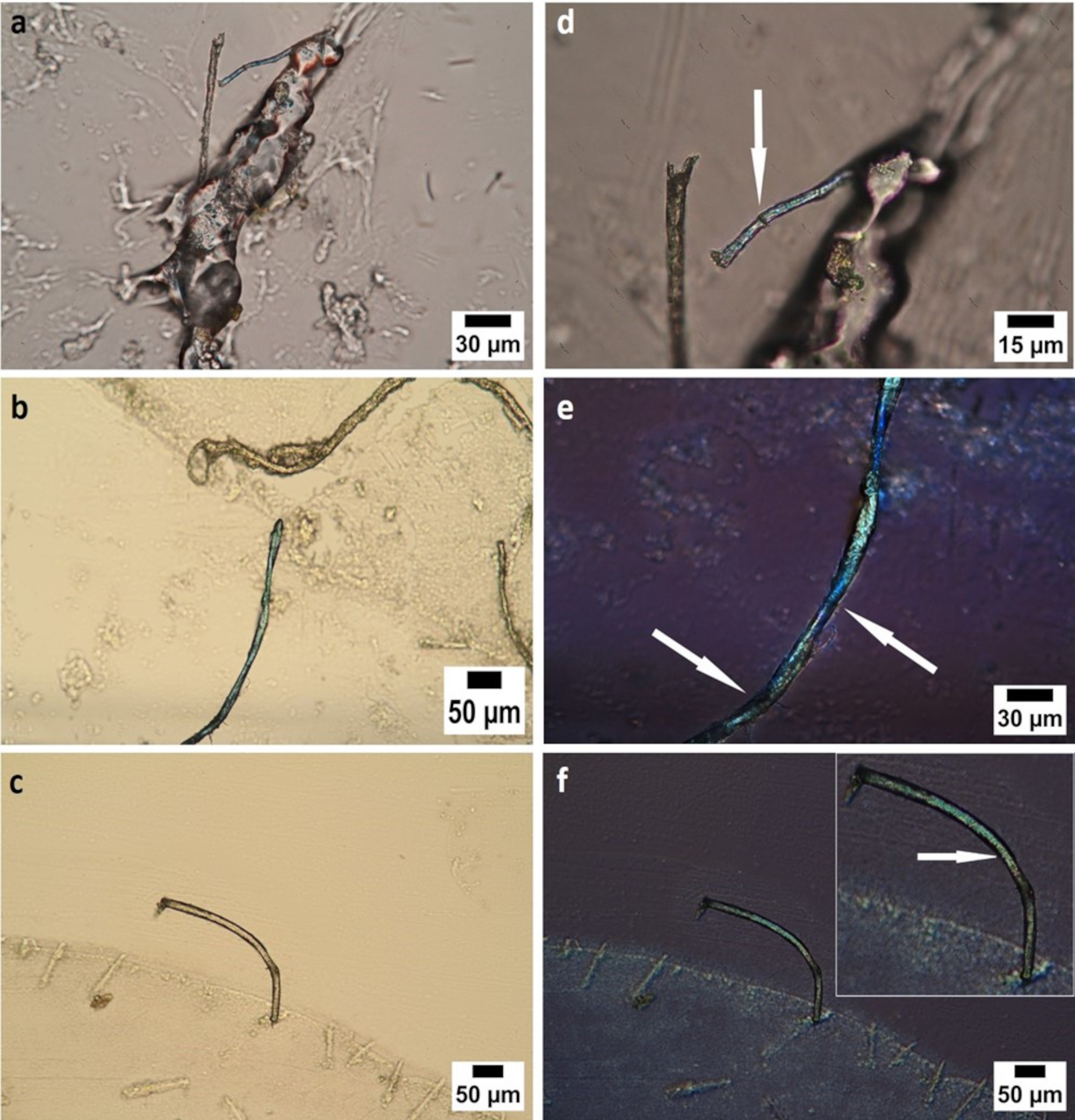

Supplement: S7 Fig — Imaging of blue and non-coloured fibres from replicative experiments on I. tinctoria by OM bright field (a-c) and polarised light (d-f). White arrows indicate kink-bands/dislocations along the length of the fibres. (FIG) [file pone.0321262.s011.tif]

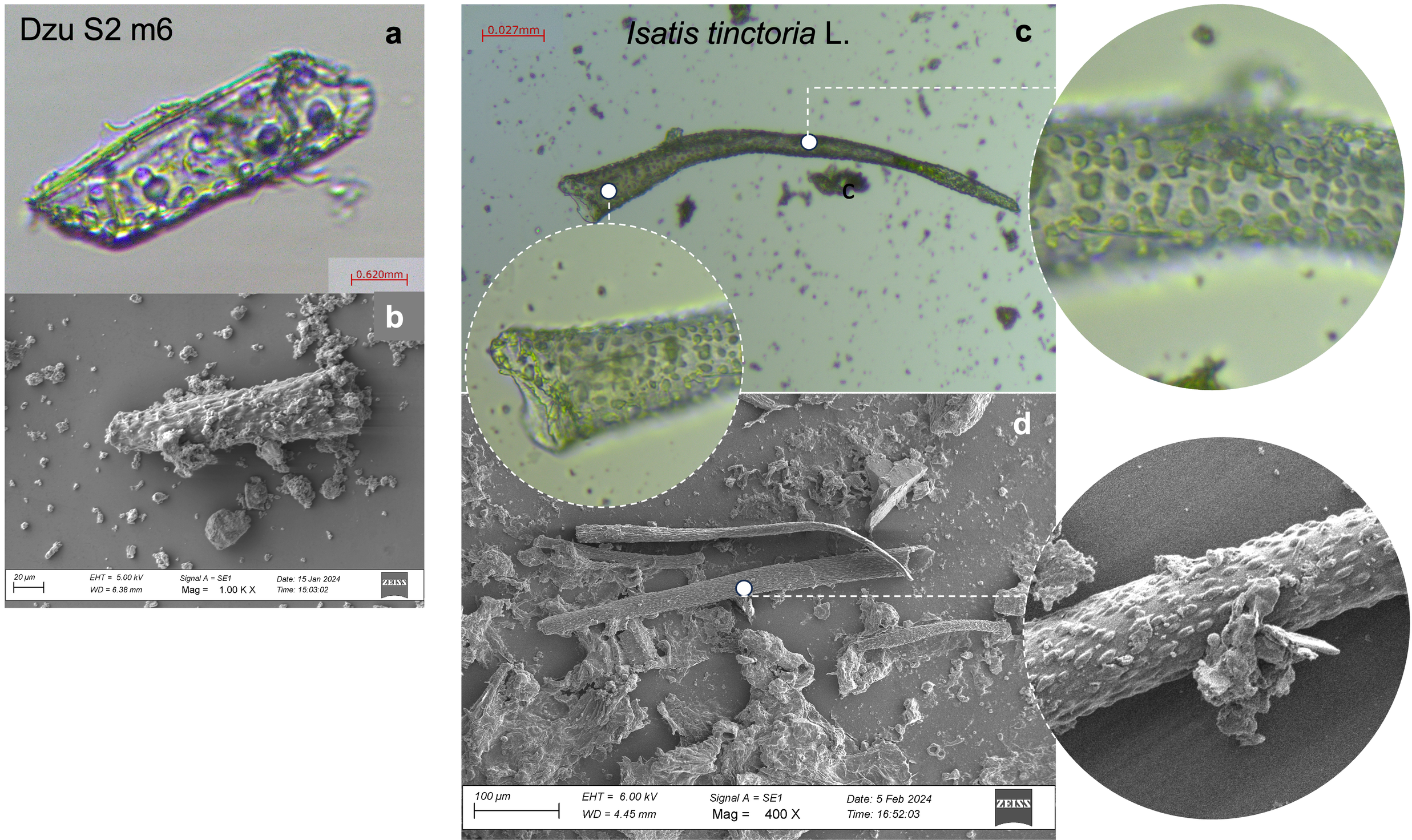

Supplement: S8 Fig — Imaging of trichomes (plant hairs): note the characteristic bumps on I. tinctoria trichomes. (a, b) archaeological micro-residues; (c, d) modern I. tinctoria leaves. The micrographs were acquired with bright field OM (a, c) and SEM (b, d). (FIG) [file pone.0321262.s012.tif]

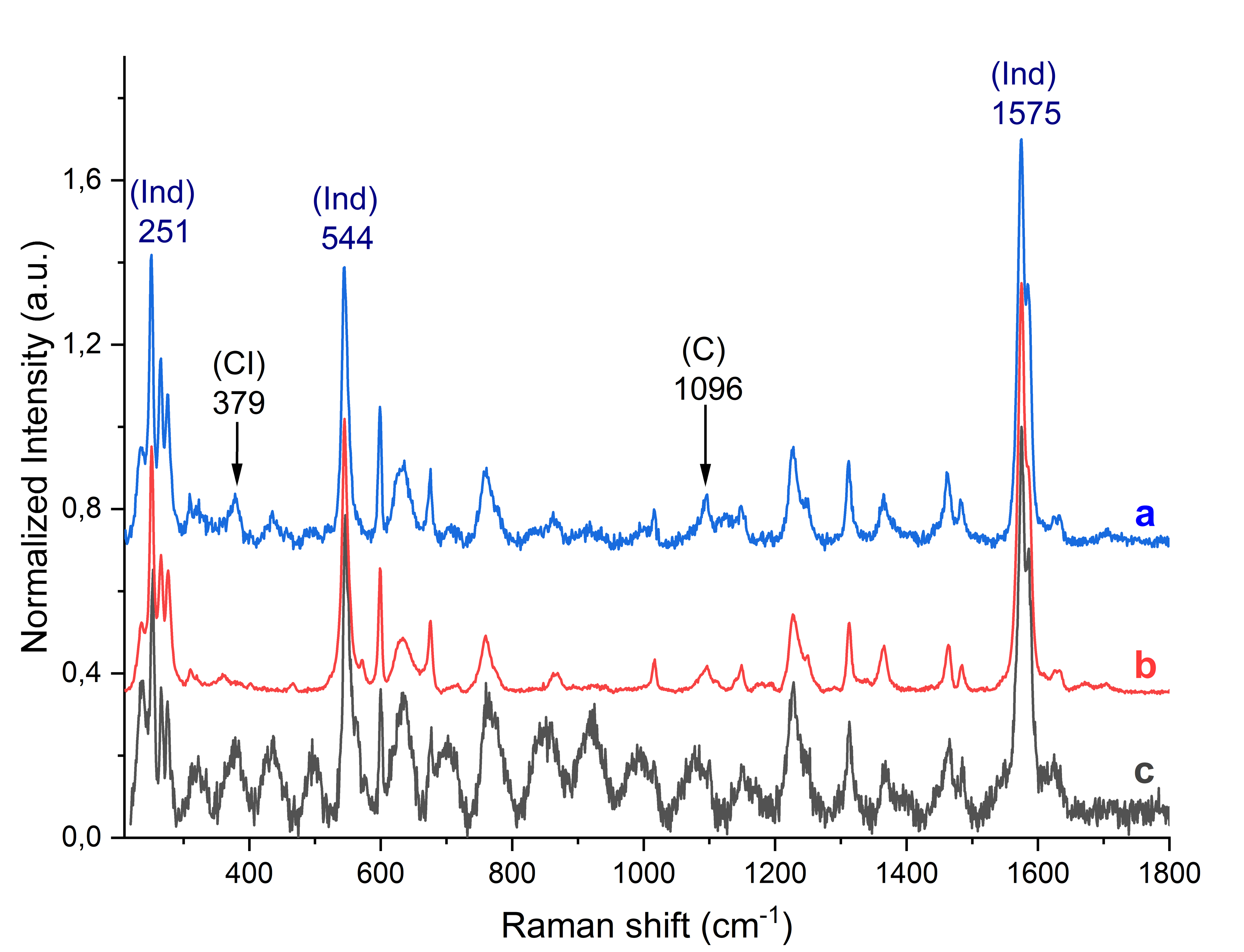

Supplement: S9 Fig — Modern reference: Normalised Raman spectra of different blue micro fibres (a, b, c) extracted from I. tinctoria leaves, excited at 785 nm and after normalisation and luminescence background removal. Characteristic bands of indigotin (Ind) and cellulose (C, CI) are indicated by their Raman shift. (FIG) [file pone.0321262.s013.tif]

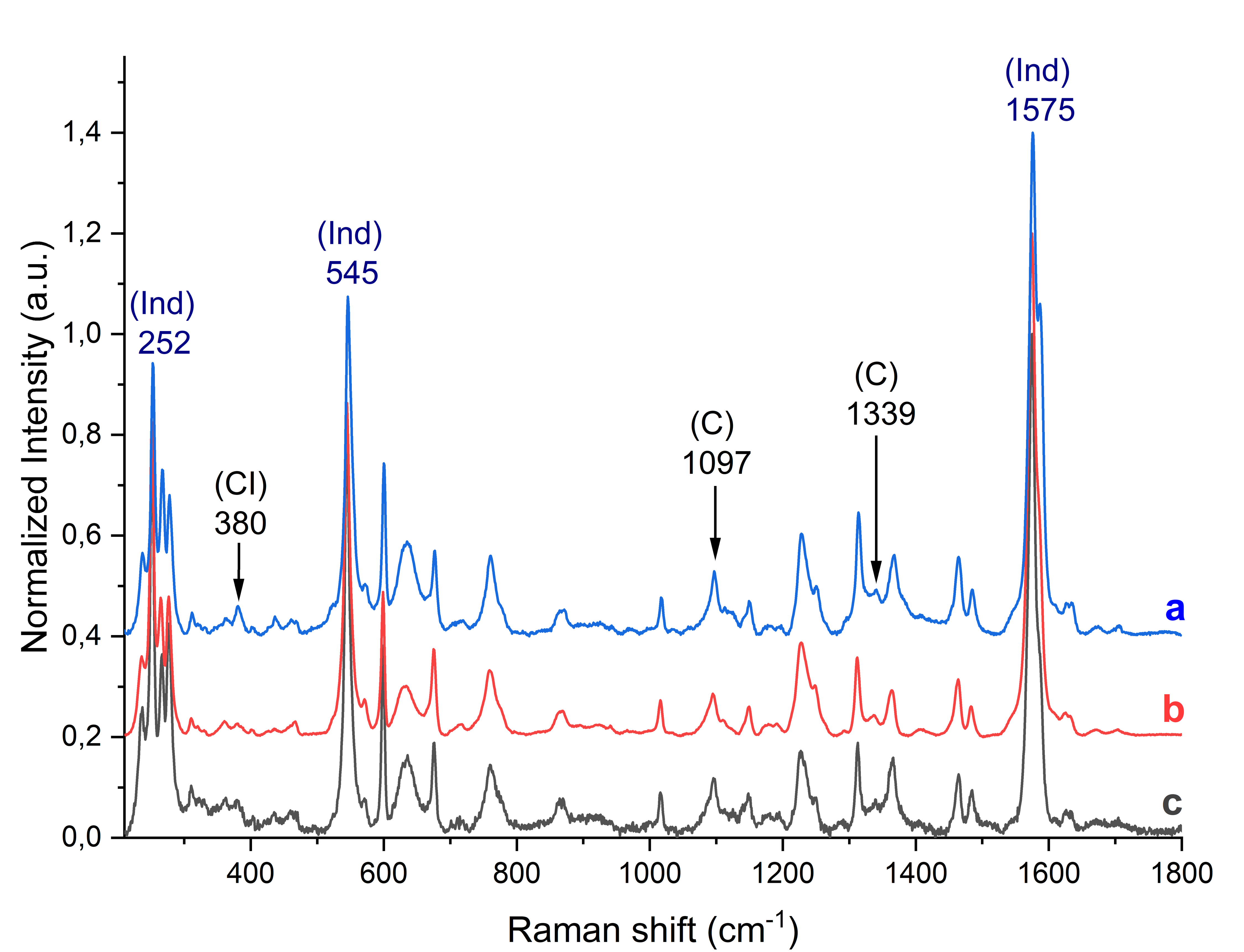

Supplement: S10 Fig — Archaeological fragments: Normalised Raman spectra of blue micro residues (a: S1 m3; b: S2 m6; c: S6 m2), excited at 785 nm and after luminescence background removal. Characteristic bands of indigotin (Ind) and cellulose (C, CI) are indicated by their Raman shift. (FIG) [file pone.0321262.s014.tif]

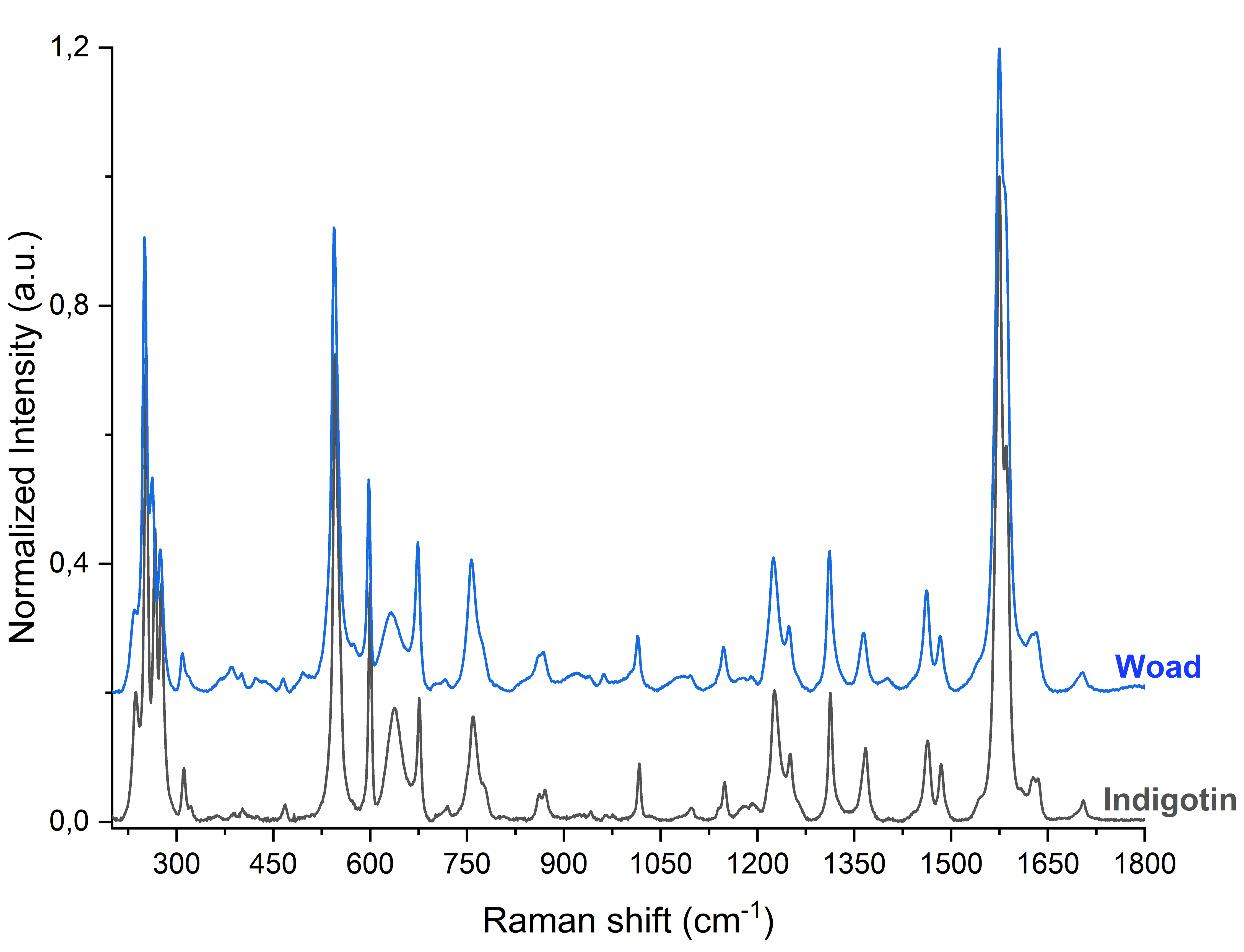

Supplement: S11 Fig — Normalised Raman spectra of natural woad dye extracted from modern I. tinctoria leaves according to hot-water extraction (see S1 File), compared with synthetic indigotin standard. Both were excited at 785 nm and they are shown after luminescence background removal. (FIG) [file pone.0321262.s015.tif]

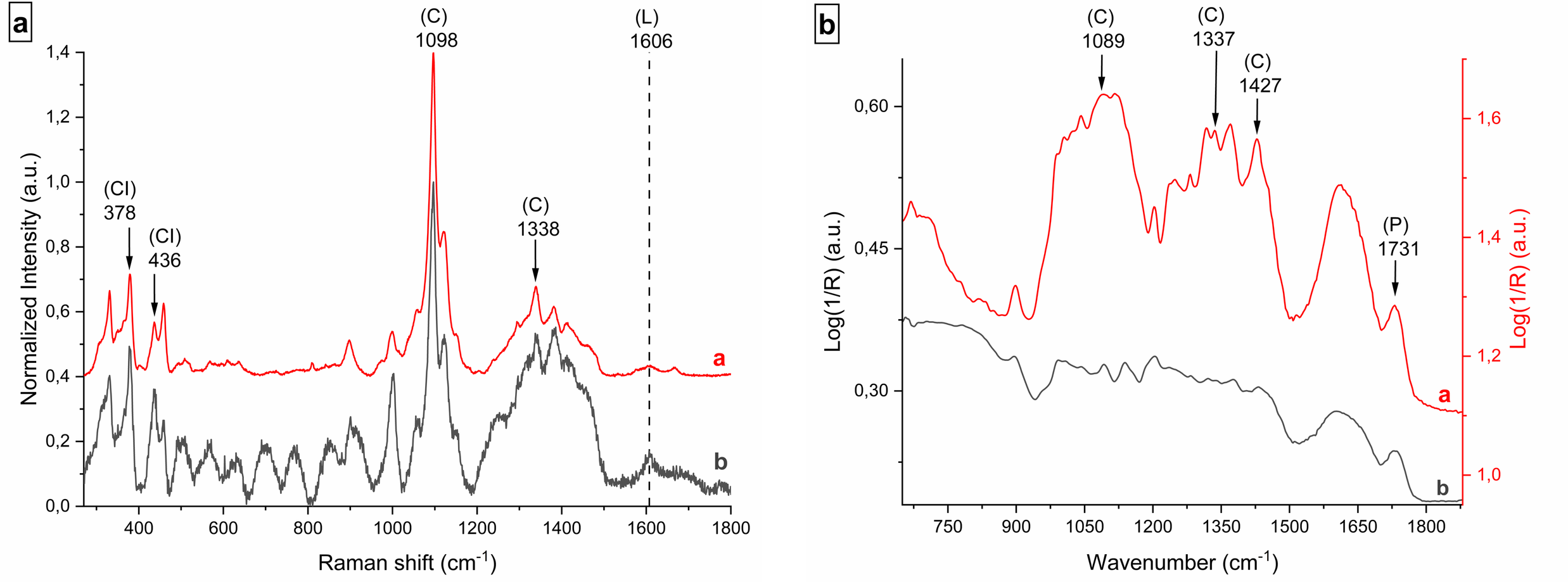

Supplement: S12 Fig — Normalised Raman after luminescence background removal (panel a), and reflectance FTIR (panel b) spectra of non-coloured micro fibres collected from modern I. tinctoria leaves. Characteristic bands of cellulose (C), including its polymorph I (CI), pectin (P) and lignin (L) are indicated by their Raman shift and wavenumber. (FIG) [file pone.0321262.s016.tif]

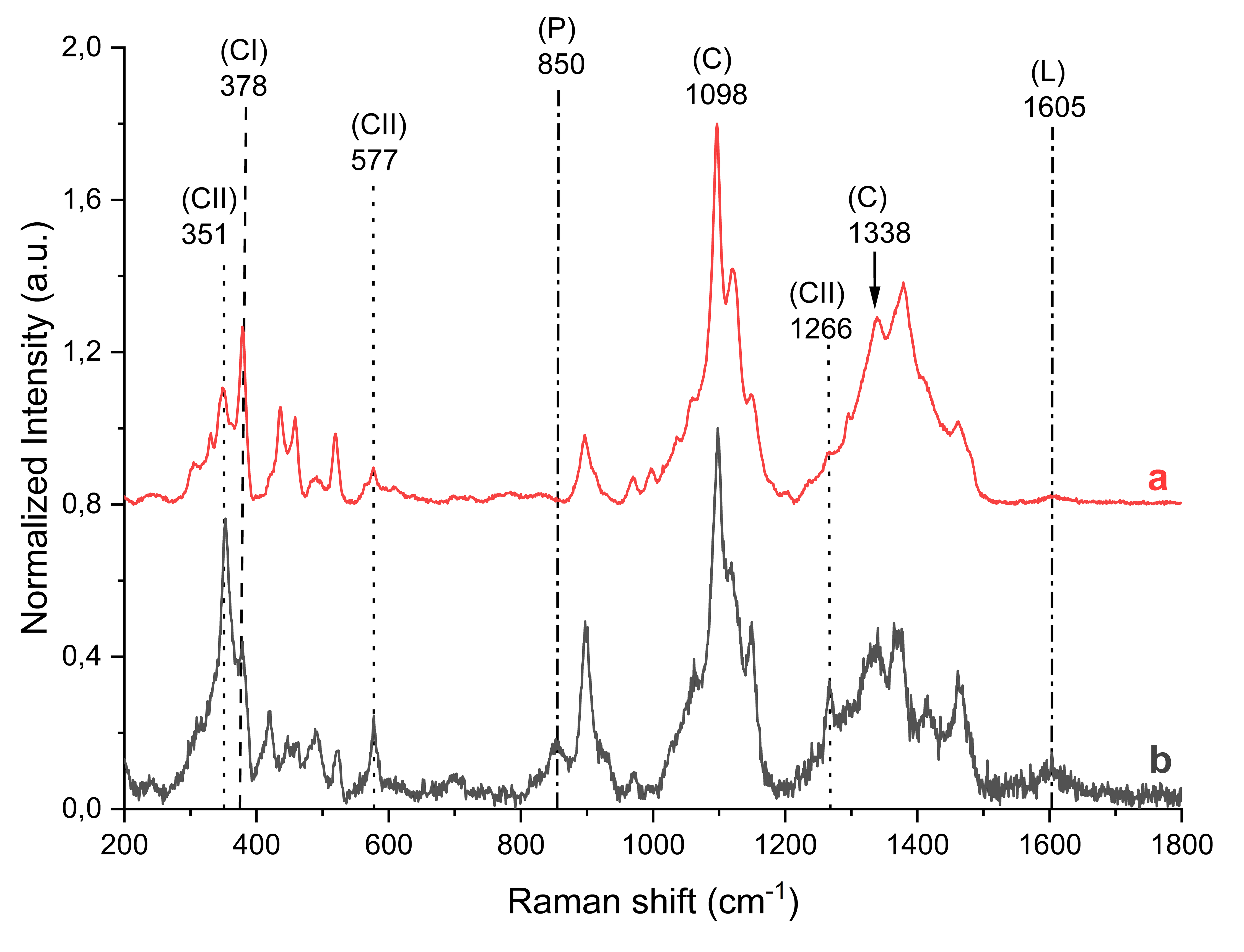

Supplement: S13 Fig — Normalised Raman spectra of archaeological non-coloured micro fragments from Dzu S6 m3 (a) and Dzu S1 m7 (b), reprised from Fig 6, excited at 785 nm and after luminescence background removal. Characteristic bands of cellulose (C), including its polymorphs I (CI) and II (CII), pectin (P) and lignin (L) are indicated by their Raman shift. (FIG) [file pone.0321262.s017.tif]

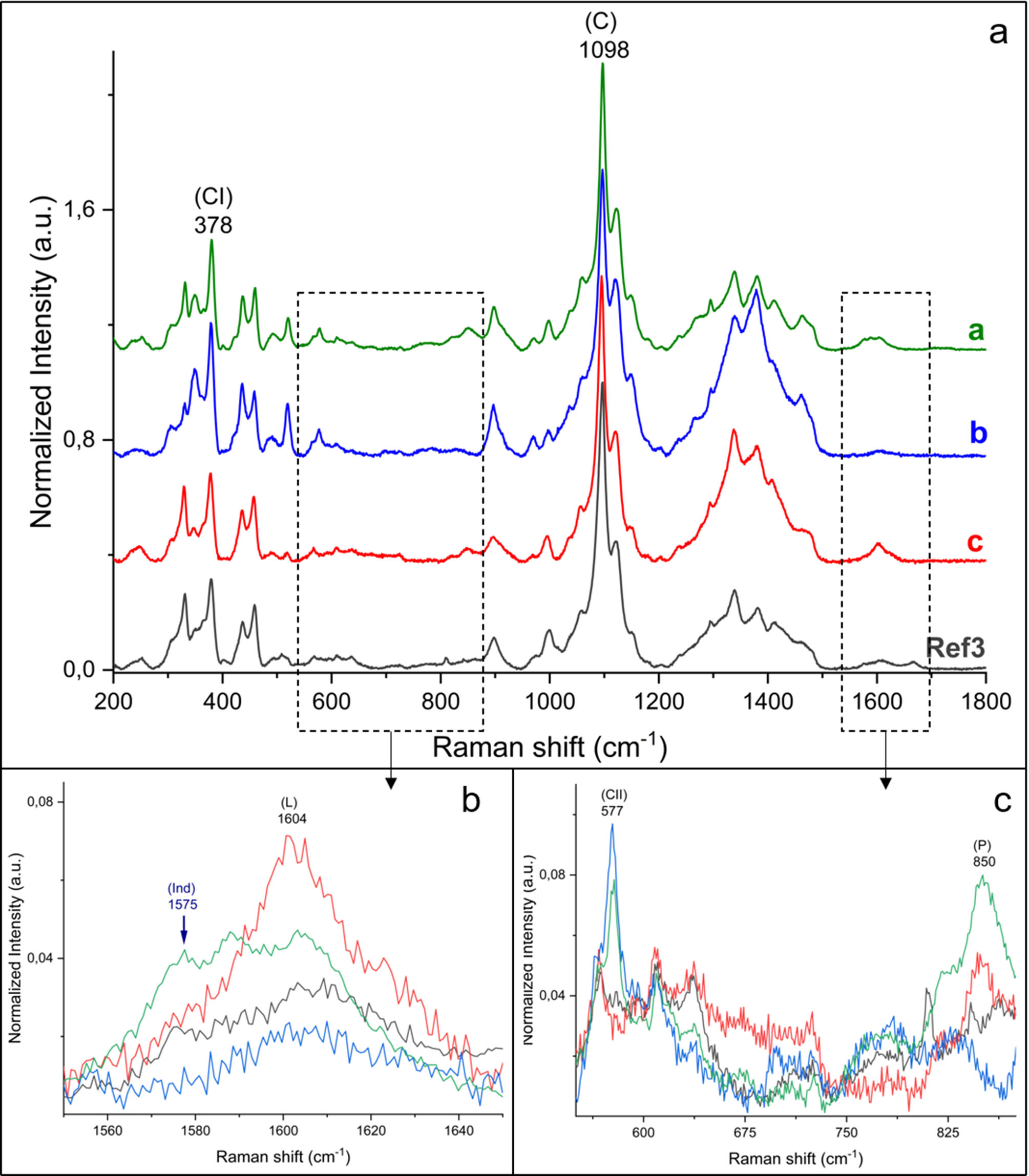

Supplement: S14 Fig — µ-Raman spectra of blue and non-coloured archaeological fragments. (a): spectrum of a non-coloured region of a blue residue; spectra b and c of non-coloured residues; spectra Ref3 of non-coloured I. tinctoria fibre obtained from the processing of modern leaves (reported as reference). (b and c): zoomed region of interest of the spectra reported in (a). This figure supports Fig 6 d of the main text. (FIG) [file pone.0321262.s018.tif]

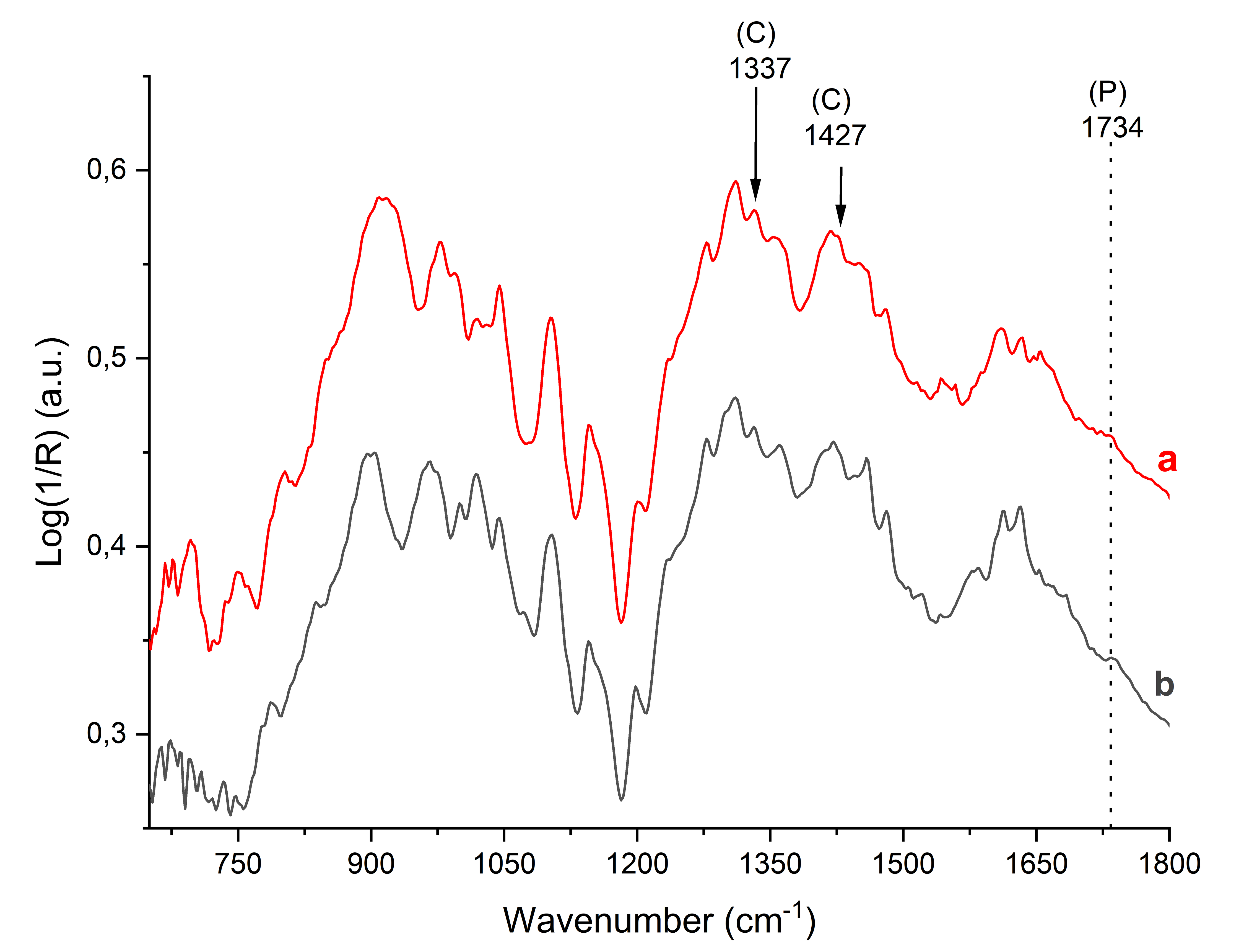

Supplement: S15 Fig — Reflectance micro-FTIR spectra of archaeological blue fragments from Dzu S1 m3 (a) and Dzu S2 m6 (b). Characteristic bands of cellulose (C) and pectin (P) are indicated by their wavenumber. (FIG) [file pone.0321262.s019.tif]

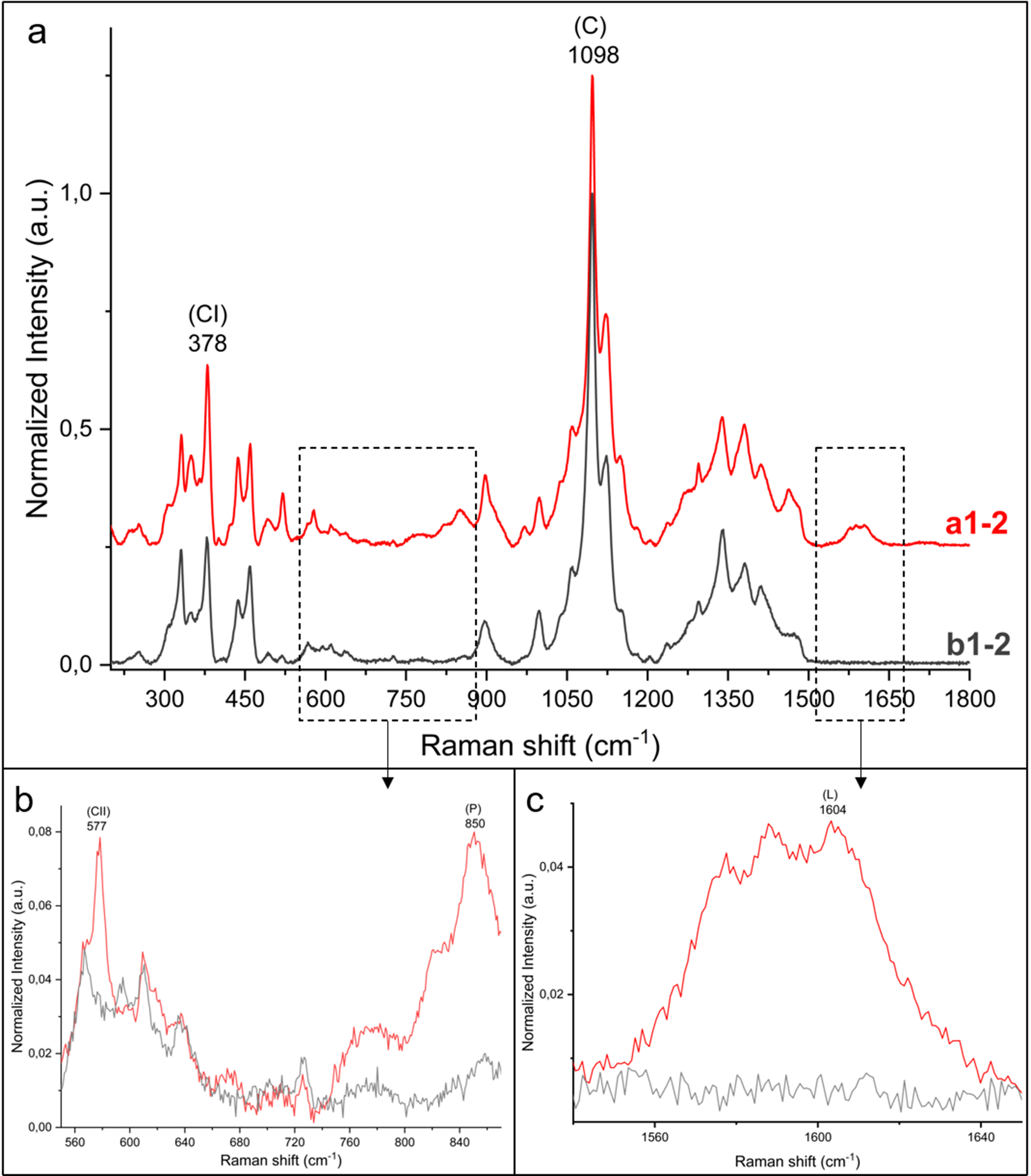

Supplement: S16 Fig — µ-Raman spectra of blue and modern fibres. (a): spectrum a1-a2 of blue archaeological residues, while spectrum b1-b2 is a modern non-coloured jeans fibres reported for comparison. (b and c): zoomed region of interest of the spectra reported in (a). This figure supports Fig 7 d of the main text. (FIG) [file pone.0321262.s020.tif]

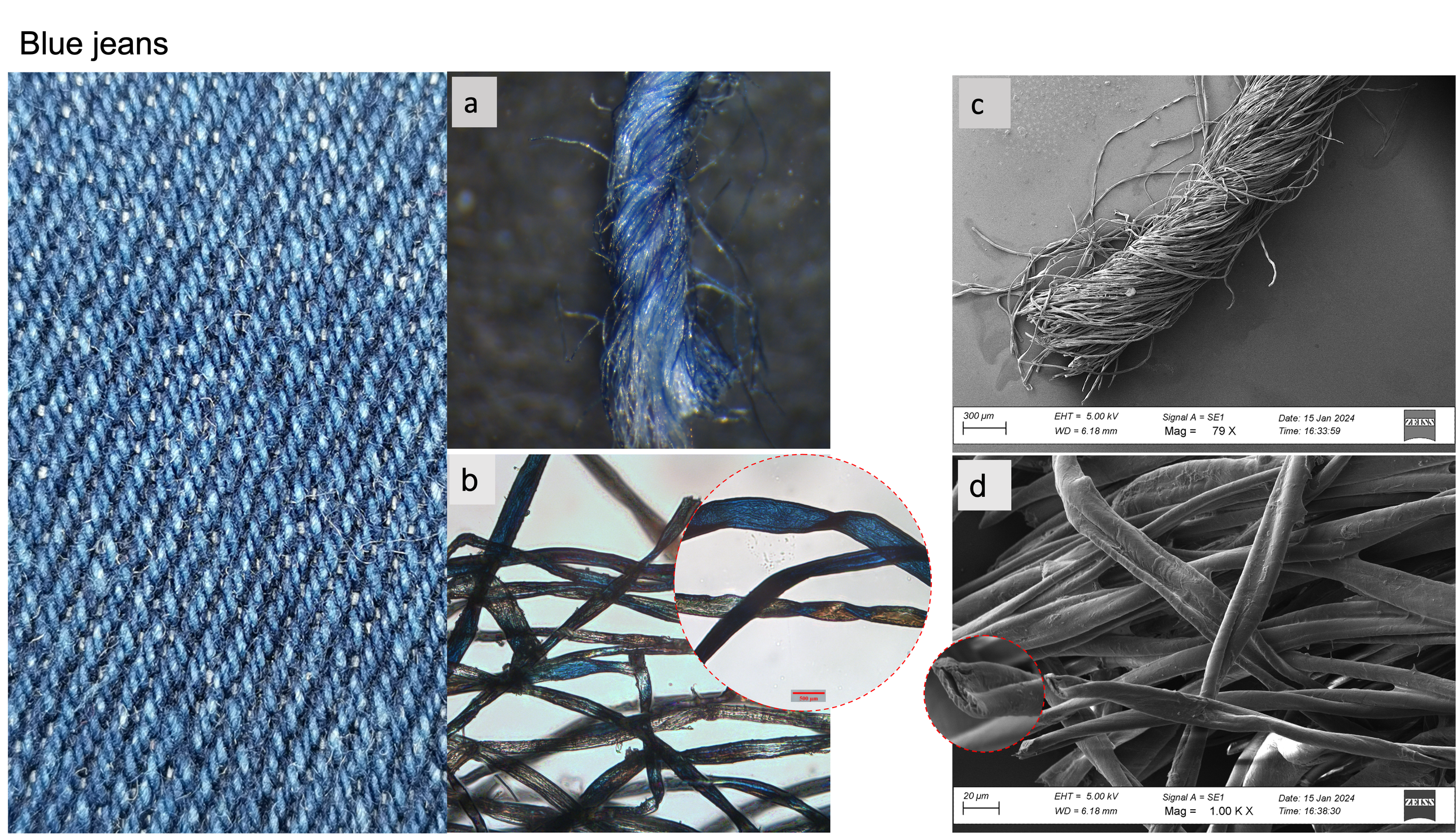

Supplement: S17 Fig — Blue jeans fabrics (Gossypium sp.) used for cross-reference to verify any potential modern contamination. (a) blue jeans fabric and detail of a thread under the stereomicroscope; (b) blue jeans fibres under OM, in the insert characteristic features of cotton are visible: ribbon-like kidney-shaped fibres, flattish in section that vary in diameter and form twists or bends along their length, a feature known as convolution; (c-d) the same thread as in (a and b), observed with SEM. Insert in (d) shows the structure of the cotton fibre. (FIG) [file pone.0321262.s021.tif]
