# Supplementary material for: Direct evidence for processing Isatis tinctoria L., a non-nutritional plant, 32–34,000 years ago
Source: PLoS One. 2025 May 9;20(5):e0321262. doi: 10.1371/journal.pone.0321262 (PMC12063890; doi:10.1371/journal.pone.0321262)
Supplement: S1 File — (PDF) [file pone.0321262.s022.pdf]

Replicative experiments

Pounding leaves for woad ball preparation (S1 Video). Shortly after cutting, 800 g of leaves were pounded using a stone pebble in a wooden mortar. The mechanical processing is characterized by a combined vertical action (such as pounding) and horizontal linear motion that shreds the leaves, repeated until a poultice is formed. During the mechanical processing, the active pestle can come into contact with the underlying wooden base, which is part of the complex system used to operate the pounding of the leaves and the subsequent collection of the poultice. It is evident that the poultice (a medium between the pestle and the wooden base) offers different resistance compared to the stone and wood. Due to its looseness, direct contact between the stone and the wooden base, whose resistance is higher than that of the poultice, can occasionally occur. This direct contact can be responsible for the striations, which may also appear as parallel linear features. Furthermore, replicative experiments have shown that the poultice tends to overflow from the direct contact area and adhere to the hand operating the stone, meaning the residues can easily spread across multiple areas, not necessarily limited to those in direct contact with the working area (Supplementary Video). The obtained poultice was then kneaded into woad balls, also known as *cocagnes,* which were left to ferment for 12 to 16 weeks, and from time to time sprinkled with water. Once dried the *cocagne* was pounded to obtain a bluish powder (S1 Video).

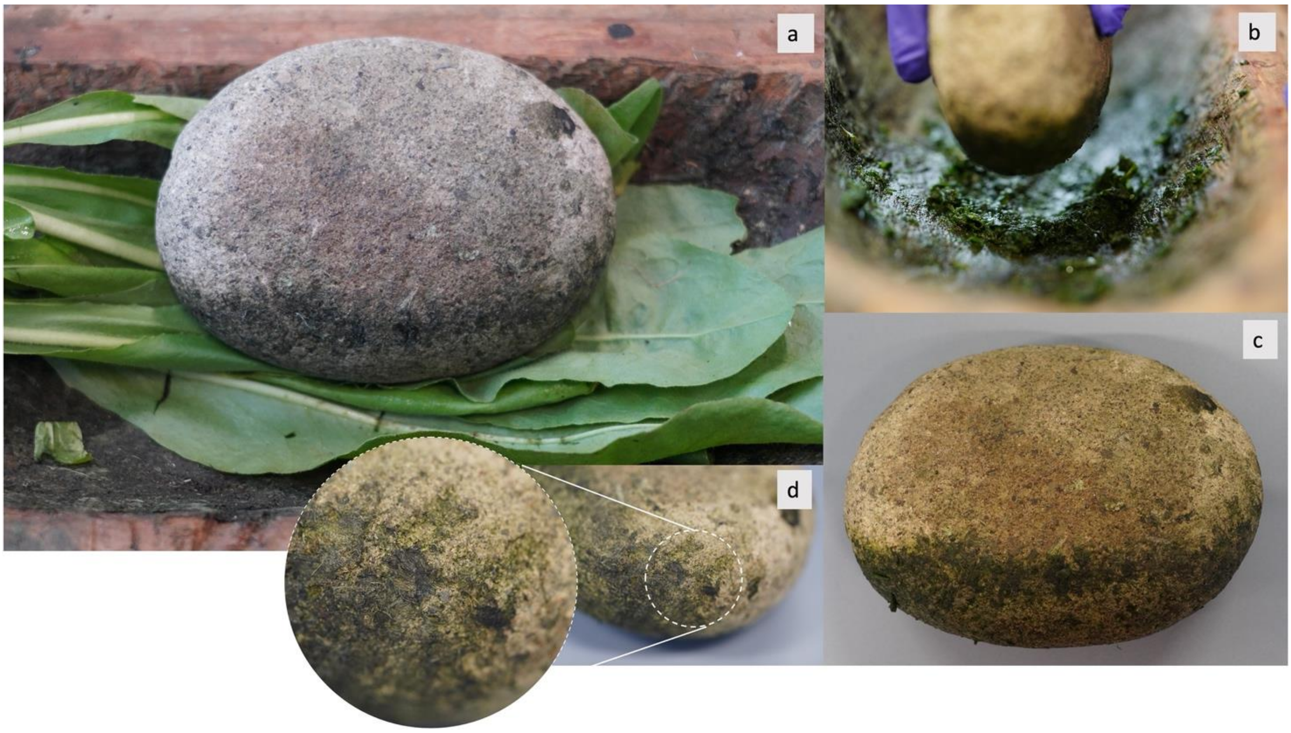

Replicative experiment setup. a: Ground stone tool, Isatis tinctoria L. leaves, wooden base; b: pounding activity; c: used pebble tool; d: I. tinctoria leaves fragments adhering to the stone surface.

Hot-water extraction (S1 Video). Another aliquot of 800 g, from the same leaves harvest, was used for hot-water extraction. The leaves were roughly shredded, soaked in a bucket or container (made out of leather or bark) with water, and the temperature was raised up to 80° C by the addition of stones, pre-heated in a fire made with *Fagus sylvatica* wood (beech). Beech was available during the early Gravettian period to hunter-gatherers living in the Caucasus, our area of interest. Beech ash is naturally high in alkali, thus, when added to the mixture, helps to reach the crucial pH of 10, fundamental to converting the indigo precursor into indigotin. Once cold, the solution was oxygenated and filtered. The insoluble indigotin precipitated during the next 12 hours. The stones used to raise the temperature were observed under the microscope and *I. tinctoria* residues and its distribution on the surface were studied.

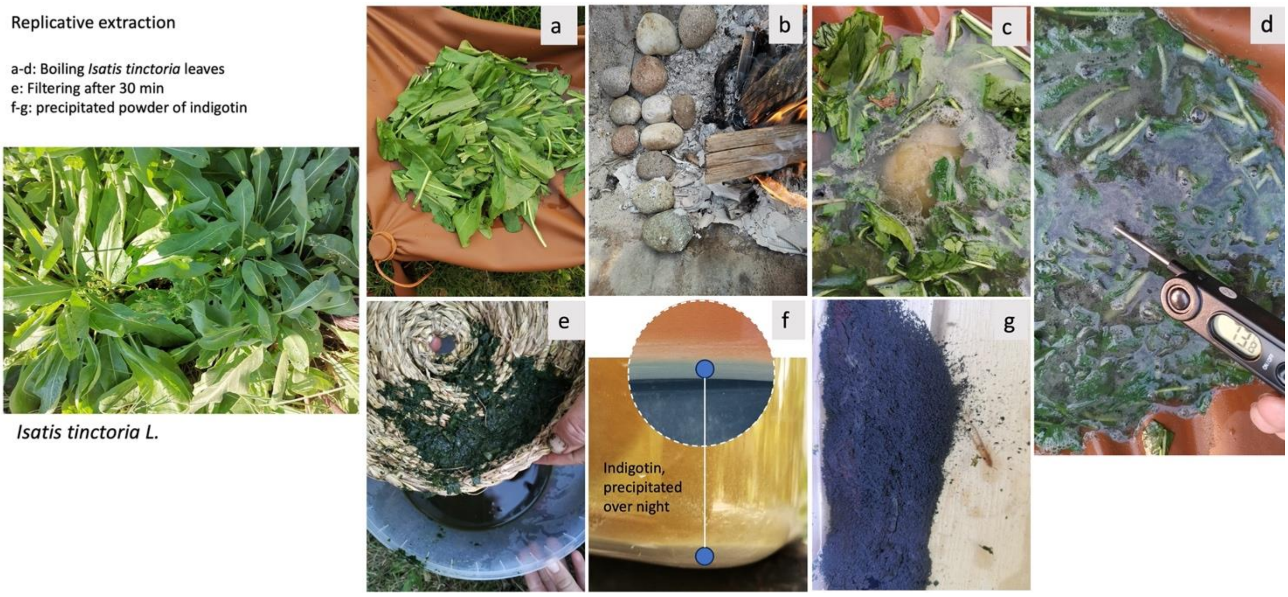

Replicative experiment setup. a: Isatis tinctoria L. broken leaves in a leather container; b: heating up the stones; fire made with beech wood; c: boiling pebble; d: the temperature does not exceed 80 Celsius degrees; g: filtering the solution after 30 minutes; f: indigotin precipitated over the night; g: powder of indigotin.
